# Replacement of dietary fish meal with *Clostridium autoethanogenum* meal on growth performance, intestinal amino acids transporters, protein metabolism and hepatic lipid metabolism of juvenile turbot (*Scophthalmus maximus* L.)

**DOI:** 10.3389/fphys.2022.981750

**Published:** 2022-08-24

**Authors:** Jichang Zheng, Wencong Zhang, Zhijie Dan, Yanwen Zhuang, Yongtao Liu, Kangsen Mai, Qinghui Ai

**Affiliations:** ^1^ Key Laboratory of Aquaculture Nutrition and Feed (Ministry of Agriculture and Rural Affairs), Key Laboratory of Mariculture (Ministry of Education), Ocean University of China, Qingdao, China; ^2^ Laboratory for Marine Fisheries Science and Food Production Processes, Pilot National Laboratory for Marine Science and Technology (Qingdao), Qingdao, China

**Keywords:** *Scophthalmus maximus* L., *Clostridium autoethanogenum* meal, growth performance, amino acids transporter, protein metabolism, lipid metabolism

## Abstract

*Clostridium autoethanogenum* meal (CAM) is a novel single-cell protein, which is produced from bacteria using carbon monoxide (CO) as sole carbon source. To evaluate the efficiency of CAM as an alternative for dietary fish meal, a 56-days growth experiment was performed on juvenile turbot (*Scophthalmus maximus* L.) with initial average weight of 9.13 ± 0.02 g. Six iso-nitrogenous (crude protein, 51.0%) and iso-lipidic (crude lipid, 11.5%) diets were formulated with 0%, 15%, 30%, 45%, 60% and 80% dietary fish meal protein substituted by CAM protein, which were designated as CAM0 (the control group), CAM15, CAM30, CAM45, CAM60 and CAM80, respectively. Results showed that no significant differences were observed in survival rate (over 97.50%) among different dietary treatments (*p >* 0.05). The specific growth rate (SGR) was not significantly affected when replacement levels of dietary fish meal with CAM were less than 45% (*p >* 0.05). The feed intake (FI) was significantly linear reduced with increasing dietary CAM (*p <* 0.05), whereas no significant differences were observed in feed efficiency ratio (FER), protein efficiency ratio (PER) and protein retention (PR) among different dietary treatments (*p >* 0.05). With increasing dietary CAM, lipid retention (LR) and carcass lipid tended to be increased in both significantly linear and quadratic patterns (*p <* 0.05). The apparent digestibility coefficient (ADC) of crude protein and some essential amino acids, including threonine, valine, lysine, histidine and arginine, showed significantly linear increase with increasing dietary CAM (*p <* 0.05). Furthermore, with the increase of dietary CAM, the gene expression of intestinal peptide and amino acids transporters was first up-regulated and then down-regulated with significantly quadratic pattern (*p <* 0.05), peaking in fish fed with diets CAM30 or CAM45, which was similar to the expression of genes related protein degradation in muscle. For genes related to protein metabolism in liver and muscle, the expression of mammalian target of rapamycin (*mtor*) was not significantly affected by dietary CAM, while the general control nonderepressible 2 (*gcn2*) tended to be first up-regulated and then down-regulated with significantly quadratic pattern (*p* < 0.05). Apart from that, the lipid metabolism of turbot was also affected by high dietary CAM, evidenced by increased expression of hepatic genes related to lipogenesis as well as reduced expression of genes related to lipid oxidation and lipid transport. In conclusion, CAM can replace up to 45% fish meal protein in diet for juvenile turbot without significantly adverse effects on growth performance. But excessive dietary CAM would result in significant growth reduction, and excessive lipid deposition may also occur in fish fed diets with high levels of CAM.

## Introduction

Over the past few decades, the rapidly expanding aquaculture industry has greatly increased demand for fish meal. Combined with the rising price of fish meal and limited fishery resources, it is necessary to search for alternative protein sources applied in aquafeeds (Natale et al., 2013). Recently, the research on alternative protein sources mainly focused on vegetable protein and their fermentation treatment (Dossou et al., 2018; Ismail et al., 2020; Iqbal et al., 2021; Rahimnejad et al., 2021) as well as animal protein (Moutinho et al., 2017; Medard et al., 2018; Sabbagh et al., 2019; Tazikeh et al., 2020) for their lower price. However, the defects of poor palatability, low digestibility and imbalanced amino acids profile limit their further application in aquafeeds. Especially, the antinutritional factors present in plant protein, such as trypsin inhibitor, phytic acid and gossypol, have negative effects on the growth performance and health of aquatic animals (Bian et al., 2017; Olukomaiya et al., 2020).

Given the above shortcomings of vegetable and animal protein, researchers have been making progress towards developing novel protein source applied in aquafeeds, especially bacterial protein. The production of bacterial protein is characterized by high efficiency, weatherproof and less land occupation (Davies and Wareham, 1988; Øverland et al., 2010; Biswas et al., 2020). Among the bacterial protein currently being developed, *Clostridium autoethanogenum* protein has gradually attracted attention due to rapidly expanding production capacity. *Clostridium autoethanogenum* (Gram-positive) can use CO as carbon source and ammonia as nitrogen source to produce ethanol and protein (i.e. *Clostridiumauto ethanogenum* meal, CAM) after processes, including anaerobic fermentation, distillation, centrifugation and spray drying (Wei et al., 2018; Chen et al., 2019). According to previous statistics, for every 36,000 tonnes of CO consumed, 10,000 tonnes of ethanol and 1,500 tonnes of CAM can be produced (Abrini et al., 1994; Wei et al., 2018), indicating industrial waste CO can be well converted into economically valuable ethanol and protein. For the nutrients composition, CAM contained approximately 86.22% crude protein and 2.11% crude lipid on a dry matter basis, and essential amino acids content in CAM was higher than that in fish meal except histidine and arginine. Recently, complete genome sequence of *Clostridium autoethanogenum* has been obtained and no toxic genes were found (Humphreys et al., 2015; Utturkar et al., 2015), which preliminarily proved the CAM safety in theory. To evaluate the potential of CAM as feed protein source, only a few experiments were carried out on aquatic animals, where CAM can replace up to approximately 30%, 42.8% and 58.2% of fish meal in the diet of pacific white shrimp (*Litopenaeus vannamei*) (Yao et al., 2022), black sea bream (*Acanthopagrus schlegelii*) (Chen et al., 2019) and largemouth bass (*Micropterus salmoides*) (Yang P. et al., 2021), respectively, without significantly adverse effects on growth performance. Also, the growth of grass carp (*Ctenopharyngodon idellus*) was improved by feeding diets with 5% CAM (Wei et al., 2018). Apart from that, the antioxidant capacity of Jian carp (*Cyprinus carpio* var. Jian) and tilapia (*Oreochromis niloticus*) would be improved when dietary soybean meal was replaced by CAM (Li et al., 2021; Maulu et al., 2021). The liver and intestine health of largemouth bass could be improved by replacing up to 50% dietary fish meal with CAM (Ma et al., 2022).

Turbot (*Scophthalmus maximus* L.) is one of commercially important carnivorous economic fish, which has been widely farmed in northern China (Bian et al., 2017). However, turbot has a high dietary protein requirement (approximately from 50 to 65%) and most of the protein comes from fish meal (Lee et al., 2003; Cho et al., 2005). Therefore, exploring novel protein sources is necessary to maintain the sustainable development of turbot farming. To our knowledge, studies on CAM as substitution for dietary fish meal are few and have not been performed on turbot. Moreover, previous works on CAM replacing dietary fish meal did not investigate the reasons for growth reduction of aquatic animals fed with excessive dietary CAM.

Thus, this study was conducted to have integrated evaluation of substituting dietary fish meal with CAM on turbot, so as to determine the optimal replacement level and investigate the reasons why high dietary CAM would reduce turbot growth.

## Methods and materials

### Experimental diets

The CAM used in the present study was supplied by Beijing Shoulang Biotechnology Co., Ltd., (Beijing, China). Six iso-nitrogenous (crude protein, 51.0%) and iso-lipidic (crude lipid, 11.5%) diets were formulated with 0%, 15%, 30%, 45%, 60% and 80% dietary fish meal protein substituted by CAM protein, which were named CAM0 (the control group), CAM15, CAM30, CAM45, CAM60 and CAM80, respectively (Table 1). For the deficiencies of histidine and arginine in CAM, L-histidine and L-arginine were added into the CAM-containing diets to keep the amino acids composition similar to that of the control diet. Diets were also supplemented with yttrium oxide (Y_2_O_3_) at the level of 0.1% for digestibility analysis.

**TABLE 1:** Formulation, proximate composition and amino acids profile of experimental diets (% dry matter).

Ingredients	Diets^e^					
	CAM0	CAM15	CAM30	CAM45	CAM60	CAM80
Brown fish meal^a^	60.00	51.00	42.00	33.00	24.00	12.00
*Clostridium autoethanogenum* meal^a^	0.00	7.48	14.97	22.45	29.93	39.91
Wheat gluten meal^a^	4.09	3.51	2.93	2.34	1.76	0.98
Wheat meal^a^	19.87	21.04	22.19	23.36	24.50	26.06
Fish oil	3.89	4.57	5.25	5.94	6.63	7.55
Soy lecithin	2.00	2.00	2.00	2.00	2.00	2.00
Squid visceral meal^a^	4.00	4.00	4.00	4.00	4.00	4.00
Vitamin premix^b^	1.50	1.50	1.50	1.50	1.50	1.50
Vitamin C^b^	0.50	0.50	0.50	0.50	0.50	0.50
Mineral premix^c^	1.50	1.50	1.50	1.50	1.50	1.50
L-histidine	0.00	0.10	0.21	0.31	0.41	0.55
L-arginine	0.00	0.15	0.30	0.45	0.61	0.81
Taurine	0.50	0.50	0.50	0.50	0.50	0.50
Choline chloride	0.30	0.30	0.30	0.30	0.30	0.30
Calcium propionate	0.10	0.10	0.10	0.10	0.10	0.10
Ethoxyquinline	0.05	0.05	0.05	0.05	0.05	0.05
Ca(H_2_PO_4_)_2_	0.50	0.50	0.50	0.50	0.50	0.50
Yttrium oxide	0.10	0.10	0.10	0.10	0.10	0.10
Sodium alginate	0.10	0.10	0.10	0.10	0.10	0.10
Attractants^d^	1.00	1.00	1.00	1.00	1.00	1.00
Feed price (USD/kg)^f^	1.63	1.59	1.55	1.51	1.47	1.41
Proximate composition						
Moisture (%)	4.64	4.36	4.54	5.11	5.48	4.92
Crude protein (%/dry matter)	51.54	51.41	51.17	51.35	51.18	51.01
Crude lipid (%/dry matter)	12.11	11.71	11.49	11.58	11.74	11.00
Ash (%/dry matter)	11.63	11.32	10.68	9.25	8.21	6.51
Essential Amino acids (%/dry matter)^g^						
Threonine	2.16	2.34	2.27	2.30	2.36	2.35
Valine	2.41	2.55	2.55	2.50	2.83	2.89
Methionine	1.25	1.31	1.32	1.28	1.27	1.40
Isoleucine	2.10	2.30	2.39	2.25	2.79	2.96
Leucine	3.90	3.98	3.97	3.97	3.97	3.92
Phenylalanine	2.39	2.37	2.32	2.32	2.37	2.36
Lysine	3.74	4.03	4.06	3.95	4.37	4.48
Histidine	1.56	1.54	1.48	1.51	1.53	1.48
Arginine	2.80	2.93	2.90	2.91	2.93	2.97
Non-essential amino acids (%/dry matter)						
Aspartic acid	4.32	4.76	4.65	4.64	5.04	5.06
Serine	2.00	2.06	2.02	2.03	1.99	1.98
Glutamic acid	7.60	7.50	7.42	7.61	7.33	6.77
Glycine	3.27	3.24	3.13	3.19	3.12	2.93
Alanine	3.20	3.18	3.18	3.22	3.10	3.03
Cysteine	0.50	0.50	0.50	0.50	0.48	0.50
Tyrosine	1.67	1.73	1.74	1.71	1.82	1.84
Proline	2.39	2.38	2.37	2.36	2.23	2.09

a Brown fish meal (dry matter, 91.99%, crude protein, 71.69% dry matter, crude lipid, 9.37% dry matter); Wheat gluten meal (dry matter, 93.62%, crude protein, 80.52% dry matter, crude lipid, 1.16% dry matter); Wheat meal (dry matter, 88.20%, crude protein, 18.52% dry matter, crude lipid, 1.54% dry matter); Squid visceral meal (dry matter, 90.70%, crude protein, 37.84% dry matter, crude lipid, 7.73% dry matter). These ingredients were provided by Great seven Bio-tech (Qingdao, China). *Clostridium* autoethanogenum meal (dry matter, 92.05%, crude protein,86.22% dry matter, crude lipid, 1.96% dry matter) was provided by Beijing Shoulang Biotechnology Co., Ltd. (Beijing, China).

b Vitamin premix (mg/kg diet): retinol acetate, 32; cholecalciferol, five; alpha-tocopherol, 240; thiamin, 25; riboflavin, 45; pyridoxine HCl, 20; vitamin B12, 10; pantothenic acid, 60; folic acid, 20; niacin, 200; biotin, 60; inositol, 800; microcrystalline cellulose, 13473. Vitamin C was supplied in the form of vitamin c polyphosphate.

c Mineral premix (mg/kg diet): MgSO_4_·7H_2_O, 1200; CuSO_4_·5H_2_O, 10; FeSO_4_·H_2_O, 80; ZnSO_4_·H_2_O, 50; MnSO_4_·H_2_O, 45; CoCl_2_.6H_2_O, 50; Na_2_SeO_3_, 20; H_2_CaIO_4_, 60; zeolite powder, 13485.

d Attractants: glycine betaine: DMPT: glycine: alanine: inosine-5-diphosphate trisodium salt = 4: 2: 2: 1: 1.

e CAM0 was the control group, CAM15, CAM30, CAM45, CAM60 and CAM80 were replacement groups formulated by replacing 15%, 30%, 45%, 60% and 80% of fish meal in the control group.

f Feed price was converted from CHY to USD with the exchange rate of 1: 6.5.

g *No tryptophan was detected because of acid hrdrolysis*.

All raw ingredients were crushed through 250 μm mesh. Then, the ingredients were thoroughly mixed with fish oil and soy lecithin, and water was finally blended into the mixture to produce moist dough. Feeds were then produced with an experimental granulator (EL-220, Haiyang City Huatong Machinery Co., Ltd.) and dried in ventilated oven (CT-C-1, Jiang Yin Zhou Yuan Pharmaceutical Equipment Co., Ltd.) at 45°C for 10 h. After drying, all the produced feeds were put into bags and stored at -20°C for feeding trial.

### Experimental procedure

Juvenile turbot were purchased from Rongcheng Yuyuanxiang Aquatic Product Co., Ltd., (Rongcheng, China), and the feeding trial was carried out in the indoor flow system of Haiyang Yellow Sea Aquatic Product Co., Ltd., (Yantai, China). Prior to the formal experiment, all the fish were fed with commercial feeds to adapt to the experimental conditions.

At the beginning of the feeding trial, all the fish were starved for 24 h, and fish were weighed at random for several times to estimate the average weight. Afterwards, juvenile turbot with an average of similar weight (9.13 ± 0.02 g) were selected and randomly assigned to 18 experimental fibreglass tanks (600 L). Each diet was randomly fed triplicate groups of 30 fish. Fish were manually fed to apparent satiation twice per day (08:00 and 18:00) for 56 days. After feeding, the collected remnant feeds were weighed after drying, and seawater in each tank was refreshed after excrement being cleaned up. During the experimental period, the water temperature ranged from 18 to 20°C, salinity ranged from 30 to 32‰, and dissolved oxygen maintained at 6.0–6.5 mg/L. The photoperiod was maintained on a 12:12 h (light: dark) ratio to imitate natural light.

### Sample collection

Prior to the formal feeding trial, 20 fish were randomly collected and stored at −20°C for the analysis of carcass protein and lipid. From the sixth week of the experiment, the feces were obtained by siphoning 8 h after feeding. The collected feces from each tank were pooled into the corresponding centrifuge tubes and frozen at -20°C for the analysis of feed digestibility.

At the end of the growth experiment, all the fish were fasted for 24 h. The total number and weight of fish in each tank were recorded after being anesthetized with MS-222 (Sigma-Aldrich) at a concentration of 80 mg/L. Five fish were randomly obtained from each tank and frozen at −20°C for body composition analysis. For the enzyme activity measurement and RNA extraction, dorsal muscle, liver and anterior intestine were obtained and quickly frozen in liquid nitrogen. Meanwhile, to analyze the profile of amino acids and fatty acids, dorsal muscle was obtained from remaining fish and frozen at −80°C. Blood was sampled from the caudal vein with another five fish from each tank. After 12 h of precipitation in anticoagulant-free centrifuge tubes, serum samples obtained from centrifuged blood (3,000 rpm, 10 min) were placed in liquid nitrogen for further serum index analysis. The sampled livers were placed in 4% parafor-maldehyde solution for fixation and then transferred to 75% ethanol for storage after 24 h.

### Analysis of biochemical and flesh yield

Biochemical analysis of ingredients, diets, feces and carcass were conducted following the standards methods of Association of Official Analytical Chemists (AOAC International, 1995). Moisture was measured by drying samples at 105°C for 48 h. Ash was determined by combustion in muffle furnace at 550°C for 16 h. Crude protein was measured by using the Kjeldahl method (Kjeltec TM 8400, FOSS, Sweden). The analysis of trichloroacetic acid (TCA)-soluble protein was performed according to the method suggested by Polychroniadou et al. (1999) with some modifications (samples were mixed with 15% TCA, and supernatants used for protein content analysis were obtained by centrifuge (4,000 rpm, 10 min)). Crude lipid analysis was performed by petroleum ether (boiling point, 30-60°C) extraction using Soxhlet (Buchi 36680, Switzerland). Amino acids in ingredients, diets, feces and muscle were analyzed using amino acids analyzer (L-8900, Hitachi) after acid hydrolysis (6N HCl for 24 h at 110°C). The analysis of fatty acids profile in ingredient, dorsal muscle and diets (Table 2) was performed with gas chromatograph-mass spectrometer (GC-MS, QP2010 Plus, SHIMADZU, Japan) following the method described by Li et al. (2019). For digestibility analysis, the freezing-dry diets and feces were firstly digested by nitric acid together with hydrofluoric acid, and the yttrium oxide content was measured by inductively coupled plasma-atomic emission spectrophotometer (ICP-OES, Thermo Fisher 7200).

**TABLE 2:** Fatty acids profile of experimental diets (% total fatty acids).

Fatty acids profile/(% total fatty acids)	Diets^a^					
	FM	CAM15	CAM30	CAM45	CAM60	CAM80
C14:0	5.07	5.07	5.18	5.27	5.56	5.74
C15:0	0.54	0.55	0.57	0.59	0.61	0.64
C16:0	23.99	24.14	24.63	25.10	25.24	25.83
C18:0	5.80	5.63	5.50	5.36	4.98	4.77
C23:0	0.49	0.47	0.45	0.43	0.40	0.36
C24:0	1.69	1.62	1.56	1.47	1.37	1.24
∑ SFA^b^	37.58	37.49	37.88	38.23	38.16	38.57
C16:1	4.98	4.91	4.82	4.71	4.71	4.51
C17:1	0.70	0.85	0.99	1.15	1.34	1.52
C18:1,cis	0.21	0.21	0.22	0.22	0.22	0.23
C18:1,trans	13.51	13.71	13.89	14.06	13.93	14.12
C20:1	3.96	4.44	4.90	5.40	5.65	6.36
∑ MUFA^b^	23.36	24.12	24.82	25.54	25.85	26.73
C18:2 n-6	10.41	10.56	10.69	10.76	10.99	11.11
C20:2 n-6	0.23	0.24	0.24	0.24	0.25	0.25
C20:4 n-6	0.84	0.81	0.78	0.76	0.75	0.69
∑ n-6 PUFA^b^	11.48	11.60	11.71	11.77	11.99	12.05
C18:3 n-3	1.44	1.49	1.53	1.54	1.65	1.71
C20:5 n-3	8.53	8.08	7.68	7.20	7.08	6.34
C22:6 n-3	13.89	13.12	12.50	11.80	11.33	10.43
∑ n-3 PUFA^b^	23.86	22.69	21.71	20.54	20.06	18.48
∑ SFA/∑ PUFA	1.06	1.09	1.13	1.18	1.19	1.26
∑ n-3 PUFA/∑ n-6 PUFA	2.08	1.96	1.85	1.75	1.67	1.53

a CAM0 was the control group, CAM15, CAM30, CAM45, CAM60 and CAM80 were replacement groups formulated by replacing 15%, 30%, 45%, 60% and 80% fish meal in the control group.

b SFA, saturated fatty acids; MUFA, monounsaturated fatty acids; n-6 PUFA, n-6 polyunsaturated fatty acids; n-3 PUFA, n-3 polyunsaturated fatty acids. Some fatty acids with extremely low content are not listed.

As to flesh yield analysis, the body weight was first recorded for each fish, and then the head and fins were cut off. Then, the remaining portion was heated in boiling water for 5 min to remove the flesh and obtain the bone. The flesh weight was then obtained by subtracting the weight of the head, fins and bones from the body weight.

### Analysis of serum and hepatic biochemical index

Serum and hepatic biochemical index, including high-density lipoprotein cholesterol (HDL-C), high-density lipoprotein cholesterol (LDL-C), total cholesterol (T-CHO), triglycerides (TG), glucose, glutamic oxalacetic transaminase (GOT) and glutamic-pyruvic transaminase (GPT), were analyzed by microplate reader (SpectraMax i3, Molecular Devices) according to the instructions of commercial reagent kits purchased from Nanjing Jiancheng Bioengineering Institute (Nanjing, China).

### Preparation and observation of liver sections

The paraffin sections of livers were made according to the method described by Zheng et al. (2022). The sampled liver was cut into small cubes, then dehydrated in anhydrous ethanol and xylene, and finally embedded in paraffin. Then tissues were sliced (5 μm) and stained with haematoxylin and eosin. Slices were observed and pictured via imaging microscope (CX31RTSF, Olympus, Japan).

### Total RNA extraction, cDNA synthesis and real-time quantitative polymerase chain reaction (RT-qPCR)

Total RNA was extracted from different tissues (intestine, liver and muscle) using RNAiso Plus reagent (Takara, Japan), and the integrity examination of RNA was conducted by electrophoresis of 1.2% denaturing agarose gel. The purity and concentration of obtained RNA were analyzed by Nano Drop^®^ 2000 spectrophotometer (Thermo Fisher Scientific, United States). All the extracted RNA was of high purity characterized by the ratio of 260/280 nm and 260/230 nm absorbance ranged from 1.98 to 2.06 and 2.00 to 2.22, respectively. Then RNA was reverse transcribed to cDNA by Prime Script-RT reagent Kit (Takara, Japan). The RT-qPCR was then carried out in quantitative thermal cycler (CFX96TM Real-Time System, BIO-RAD, United States) referring to the method of Zuo et al. (2012). The primer sequences of reference gene and target genes of turbot (Table 3) were synthesized according to published papers (Xu et al., 2016; Xu et al., 2017). The expression level of the target genes was normalized by the reference gene of RNA polymerase II subunit D (*rpsd*) via the method expressed as 2^−(ΔΔCT)^ (Xu et al., 2016).

**TABLE 3:** Primer sequences used for real-time quantitative PCR.

Genes^a^	Forward primer (5′–3′)	Reverse primer (5′–3′)	References (GeneBank no.)
**Peptide and amino acids transporters**			
*pept1*	GCA​TCC​ACA​CCC​AGC​AGA​AG	GTC​CTC​AGC​CCA​GTC​CAT​CC	Xu et al. (2016)
*cat2*	TGC​TGC​TGT​TCG​TGA​CCA​TCT​C	AGG​TTC​CAG​AAA​TGC​CAT​AAG​GG	Xu et al. (2016)
*b* ^*0*^ *at1*	AGA​CTC​TCA​ACA​CCT​CCG​AAG​C	AGC​CTT​TCC​TGT​GGT​CTC​AAT​CC	Xu et al. (2016)
*b* ^*0,+*^ *at*	GGG​CTT​TGG​GCT​TAT​GAT​GGA​TG	TGG​AGA​CAA​CAG​CAG​TTC​AGT​GG	Xu et al. (2016)
*pat1*	TCA​GTG​ACA​ACA​TCA​AGC​AGG​TG	GAA​GGC​GGG​CAG​GAA​GAA​GAG	Xu et al. (2016)
*asct2*	ACC​TTG​ATC​GCC​TCG​TCC​ATC	CAT​CTG​TGC​CGT​TCC​TTG​TAA​CC	Xu et al. (2016)
*snat2*	TGC​TGC​TGG​TGA​CGC​TCT​TC	TGC​TGC​TGG​TGA​CGC​TCT​TC	Xu et al. (2016)
*tat1*	TCT​CCC​ATC​GTC​AGC​GTC​TTC	CTG​CCA​GCC​GTC​ACA​ATG​C	Xu et al. (2016)
*y* ^*+*^ *lat1*	TGT​GAC​GTT​TGC​GGA​CCA​G	GAC​GGG​AGT​GTA​GCG​GAA​GAC	Xu et al. (2016)
**Genes related to protein metabolism**			
*mtor*	GCA​GGA​AGT​ACA​TGC​GGT​CT	GCT​GGT​TGG​GGT​CAT​AAG​TG	Xu et al. (2017)
*4e-bp1*	CCG​CAA​GTT​CCT​ACT​GGA​C	AGG​CTT​GCC​ATC​GTG​GTT​GT	Xu et al. (2016)
*gcn2*	ACA​GAC​GGC​GAT​CAA​CCT​C	CCT​AAA​CAG​CCT​CCA​TAA​CC	Xu et al. (2017)
*atrogin-1*	AGG​AGA​ACT​TGC​TGC​TGT​CG	AGA​TCC​AAG​CGG​TTG​AAG​G	Song et al. (2017)
*capns1-like*	CAT​GAA​GAG​ACC​GGA​CCA​GG	TAT​TTT​GGT​CCC​GTT​GCC​GT	XM_035622025.1
*atg4b*	TCA​GTG​TGG​ATG​CCC​TGA​AC	GAG​ACG​GAG​AAT​CCC​TTG​CC	XM_035648518.1
*atg12*	CTC​GGA​ACT​ACT​AGC​CGC​TG	GAG​TGT​CTC​CCA​CTG​CCT​TC	XM_035643075.1
*lc3b*	GAG​GAA​GGA​AGC​GAC​GAC​AT	GTC​CGA​GGT​TTG​AGG​CGA​A	XM_035642079.1
*cathepsin-d*	ACG​ACA​GAG​TTG​GCT​TTG​CT	GTC​AAC​TCT​CCA​ATC​TGC​TGG​A	XM_035627591.1
*calpastatin*	CTA​AAC​CCG​AGC​CCA​GCA​CA	CTA​AAC​CCG​AGC​CCA​GCA​CA	XP_035486615.1
**Genes related to lipid metabolism**			
*fas*	GGC​AAC​AAC​ACG​GAT​GGA​TAC	CTC​GCT​TTG​ATT​GAC​AGA​ACA​C	Peng et al. (2014)
*lxr*	GCC​TTT​CAG​TTC​ACC​ATC​ACA	ATC​TGA​TTT​GCT​CCT​CCG​AG	Peng et al. (2014)
*pparγ*	AAG​TGA​CGG​AGT​TCG​CCA​AGA	GTT​CAT​CAG​AGG​TGC​CAT​CA	Peng et al. (2014)
*srebp-1*	CGATCCGCACTCCAAGT	CCGCACTGCCCTGAAT	Peng et al. (2014)
*lipin1*	AGG​ACG​CTG​GTG​GTT​CTC​G	CTG​TCC​GCT​GAG​GTC​ATA​GTG	Peng et al. (2014)
*lpl*	CTCCCACGAACGCTCTAT	GCGGACCTTGTTGATGTT	Peng et al. (2014)
*ampk1α*	TGG​AGC​AGT​GGG​GTC​ATT​C	ATG​GGG​TCC​ACC​TGA​AGC​A	Peng et al. (2014)
*cpt1*	GCC​TTT​CAG​TTC​ACC​ATC​ACA	ATG​CGG​CTG​ACT​CGT​TTC​TT	Peng et al. (2014)
*mtp*	CCA​GCA​AAG​TCT​TAC​GCC​A	TAC​GCA​GAT​GAT​GAC​CCA​AC	Peng et al. (2014)
*apob-100*	TCTCACCCTCGGTCTCGG	TTC​AGG​TTT​CTC​CTC​ACA​ACG​A	Peng et al. (2014)
*hnf4α*	AGT​GCG​TGG​TGG​ACA​AAG​AC	GAG​TCG​TAC​TGG​CGG​TCG​TTG	Peng et al. (2014)
**Reference gene**			
*rpsd*	CTG​CTG​TTC​CCT​AAA​GAG​TTC​G	GAG​CCG​TGT​AGT​TCA​GGG​TCT	DQ848899.1

a *pept1*, peptide transporter 1; *cat2*, cationic amino acid transporter 2; *b^0^at1*, B^0^-type amino acid transporter 1; *b^0,+^at*, b^0,+^-type amino acid transporter; *pat1*, proton-coupled amino acid transporter 1; *asct2*, system ASC amino acid transporter-2; *snat2*, sodium-coupled neutral amino acid transporter 2; *tat1*, T-type amino acid transporter 1; *y^+^lat1*, system y^+^L amino acid transporter 1; *gcn2*, general control nonderepressible 2; *mtor*, mammalian target of rapamycin; *4e-bp1*, eukaryotic initiation factor 4E-binding protein 1; *capns1-like*, calpain small subunit 1-like; *lc3b*, microtubule associated protein 1 light chain 3 beta; *atg4b*, autophagy related 4B cysteine peptidase; *atg12*, autophagy related 12 homolog; *fas*, fatty acid synthase; *lxr*, liver X receptor; *pparγ*, peroxisome proliferator-activated receptor γ; *srebp-1*, sterol-regulatory element binding protein-1; *lpl*, lipoprotein lipase; *ampk1α*, adenosine monophosphate activated protein kinase1α; *cpt1*, carnitine palmitoyl transferase 1; *mtp*, mitochondrial trifunctional protein; *apob-100*, apolipoprotein B-100; *hnf4α*, hepatocyte nuclear factor 4α; *rpsd*, RNA polymerase II subunit D.

### Calculations and statistical analysis

The following parameters were calculated:

$$Survival\;rate\;\left(\%\right)\;=\;100\;\times \;\left(N_{t}/N_{0}\right)$$

$$Specific\;growth\;rate\;\left(SGR,\;\%/day\right)\;=\;\left(Ln\;W_{t}\;-\;Ln\;W_{0}\right)\;\times \;100/t$$

$$\text{Weight gain rate }\left(\text{WGR, \%}\right)\;=\;\left(W_{t}\;-W_{\text{0}}\right)\text{ × }100/W_{\text{0}}$$

$$\text{Feed intake }\left(\text{FI, \%BW/day}\right)\;=\;100{\mathrm{\times I}}_{\text{d}}\text{/}(\left(W_{t}\;+W_{\text{0}}\right)\text{/2})/t$$

Feed efficiency ratio (FER) = Wet weight gain (g)/dry feed fed (g)

Protein efficiency ratio (PER) = Wet weight gain (g)/dry protein fed (g)

$$\text{Protein retention }\left(\text{PR}\right)\;=\;\left(W_{t}{\mathrm{\times P}}_{t}\;-W_{\text{0}}{\mathrm{\times P}}_{\text{0}}\right)\text{/}\left(I_{\text{d}}\mathrm{\times P}\right)$$

$$\text{Lipid retention }\left(\text{LR}\right)\;=\;\left(W_{t}{\mathrm{\times L}}_{t}{\;-W}_{\text{0}}{\mathrm{\times L}}_{\text{0}}\right)\text{/}\left(I_{\text{d}}\mathrm{\times L}\right)$$

$$\text{Flesh yield }\left(\text{FY, \%}\right)\;=\;100\text{ × }\left(W_{\text{f}}{\mathrm{/W}}_{\text{t}}\right)$$

(Bugeon et al., 2010)

$${\text{Feed cost }\left(\text{USD/kg flesh}\right)\;=\text{P}}_{\text{f}}{\text{ × I}}_{\text{d}}{\text{/W}}_{\text{f}}\;$$

(Wang et al., 2021)

$${\text{Condition factor }\left(\text{CF, \%}\right)\;=\;100\text{ × body weight }\left(\text{g}\right)\text{/body length }\left(\text{cm}\right)}^{3}$$

Hepatosomatic index (HSI, %) = 100 × liver weight (g)/body weight (g)

Viscerosomatic index (VSI, %) = 100 × viscera weight (g)/body weight (g)

$$\begin{array}{l} \text{Apparent digestibility coefficient }\left(\text{ADC}\right)\text{ of dry matter }\left(\text{\%}\right)\\ =\;\left(100-\left({\text{dietary Y}}_{2}{\text{O}}_{3}{\text{/fecal Y}}_{2}{\text{O}}_{3}\right)\text{×100}\right) \end{array}$$

$$\begin{array}{l} \text{Apparent digestibility coefficient }\left(\text{ADC}\right)\text{ of nutrients }\left(\text{\%}\right)\\ =\;(100-\left({\text{dietary Y}}_{2}{\text{O}}_{3}{\text{/fecal Y}}_{2}{\text{O}}_{3}\right)\text{ × }\left(\text{fecal nutrients/dietary nutrients}\right)\text{×100}) \end{array}$$

Where *W*_*0*,_ *W*_*t*_ and *W*_*f*_ represent initial body weight, final body weight and flesh weight, respectively. BW is short for body weight. *t* is duration of the growth experiment. *I*_*d*_ is feed consumption. *P*_*0*_, *P*_*t*_ and *P* represent protein content in initial fish body, final fish body and diet, respectively. *L*_*0*_, *L*_*t*_ and *L* represent lipid content in initial fish body, final fish body and diet, respectively. *P*_*f*_ stands for feed price that is calculated based on feed ingredient cost. *N*_*0*_ and *N*_*t*_ are initial and final number of fish, respectively.

Statistical analysis of the data was performed using SPSS 17.0 software package for windows. The normality and homogeneity of the data were first analyzed, then polynomial contrasts analysis was also performed in the pattern of linear and quadratic. One-way analysis of variance (ANOVA) was performed, and significant differences among treatments were analyzed by Tukey’s multiple range test. The significance level was set at *p* < 0.05, and the results are exhibited as means ± S.E.M (standard error of means).

## Results

### Nutritional composition in fish meal and *Clostridium autoethanogenum* meal (CAM)

The crude protein content in CAM (86.22%) is higher than that in fish meal (71.69%) (Table 4), but the trichloroacetic acid (TCA)-soluble protein content in CAM (5.43%) is lower as compared with that in fish meal (15.84%). The essential amino acids contents in CAM were higher than in fish meal except histidine and arginine. The fatty acids profile (% total fatty acids) of CAM is mainly saturated fatty acids (i.e. myristic acid (C14:0) and palmitic acid (C16:0)), lacking polyunsaturated fatty acids (Table 5).

**TABLE 4:** The nutritional composition and price of fish meal and *Clostridium autoethanogenum* meal (CAM)^a^.

	Fish meal	CAM^b^
Price (USD/t)^c^	1846.15	1538.46
Moisture (%)	8.01	7.95
Crude lipid(%/dry matter)	9.05	2.11
Crude protein (%/dry matter)	71.69	86.22
TCA-soluble protein^d^ (%)	15.84	5.43
Essential amino acids (%/protein)		
Threonine	4.32	4.60
Valine	5.27	6.40
Methionine	2.94	3.36
Isoleucine	4.52	6.67
Leucine	7.60	7.47
Phenylalanine	4.23	4.21
Lysine	7.98	9.59
Histidine	3.31	1.52
Arginine	6.00	4.07
Non-essential amino acids		
Aspartic acid	8.90	10.19
Serine	3.98	3.75
Glutamic acid	14.52	11.98
Glycine	5.98	4.69
Alanine	6.60	5.74
Cysteine	0.78	0.89
Tyrosine	3.26	3.86
Proline	3.70	2.93

a Data are means of triplicate. No tryptophan was detected because of acid hrdrolysis.

b CAM was provided by Beijing Shoulang Biotechnology Co., Ltd.

c Price was converted from CHY to USD with the exchange rate of 1: 6.5.

d The percentage of protein extracted by 15% trichloroacetic acid (TCA), representing the relative content of small peptides and free amino acids in total crude protein.

**TABLE 5:** Fatty acids profile of fish meal and *Clostridium autoethanogenum* meal (CAM)^a^.

Fatty acids profile (% total fatty acids)	Fish meal	CAM^b^
C12:0	0.15	0.85
C14:0	10.06	16.39
C15:0	0.80	0.00
C16:0	37.63	79.61
C17:0	0.82	0.20
C18:0	8.17	0.06
∑SFA^c^	57.63	97.11
C16:1	5.08	0.69
C17:1	0.14	0.15
C18:1	0.02	0.12
C20:1	0.58	0.01
∑MUFA^c^	5.82	0.99
C18:2 n-6	0.98	0.04
C18:3 n-6	0.15	0.00
C20:2 n-6	1.13	0.00
C20:4 n-6	1.05	0.00
∑n-6 PUFA^c^	3.31	0.04
C18:3 n-3	0.60	0.00
C20:5 n-3	11.51	0.00
C22:6 n-3	19.44	0.02
∑n-3 PUFA^c^	31.55	0.03

a Data are means of triplicate.

b CAM was provided by Beijing Shoulang Biotechnology Co., Ltd.

c ∑ SFA, saturated fatty acids sum; ∑ MUFA, monounsaturated fatty acids sum; ∑ n-6 PUFA, n-6 polyunsaturated fatty acids sum; ∑ n-3 PUFA, n-3 polyunsaturated fatty acids sum. Including some minor components not shown.

### Growth performance, feed utilization and feed cost

There were no significant differences in survival rate (ranged from 97.78 to 100%) among different dietary treatments (*p* > 0.05) (Table 6). Growth parameters (FBW, SGR and WGR) were significantly linear reduced with increasing dietary CAM (*p <* 0.05), but no significant differences were observed among fish fed with diets CAM0, CAM15, CAM30 and CAM45 (*p* > 0.05). There were no significant differences in feed efficiency ratio (FER), protein efficiency ratio (PER) and protein retention (PR) among different dietary treatment (*p* > 0.05). While the lipid retention (LR) exhibited significantly linear and quadratic increase with increasing dietary CAM (*p <* 0.05). Feed intake (FI) and flesh yield (FY) showed significantly linear decrease with increasing dietary CAM, and significant differences occurred when fish fed with diet CAM80 as compared with the control group (*p <* 0.05), which was exactly opposite to the trend of feed cost. Hepatosomatic index (HSI) was not significantly different in replacement groups as compared with the control group, but it showed significantly linear increase with increasing dietary CAM (*p <* 0.05).

**TABLE 6:** Growth parameters, feed utilization and feed cost of juvenile turbot fed the experimental diets (Means ± S.E.M)^a^.

	Diets^b^						Polynomial contrasts		
	CAM0	CAM15	CAM30	CAM45	CAM60	CAM80	*p*-value	Linear	Quadratic
FBW^c^ (g)	52.63 ± 2.09^a^	49.32 ± 2.26^a^ ^,^ ^b^	48.21 ± 0.85^a^ ^,^ ^b^	43.61 ± 1.38^a^ ^**,**^ ^b^	42.72 ± 3.22^b^	41.32 ± 1.63^b^	0.014	0.001	0.096
Survival rate (%)	100.00 ± 0.00	100.00 ± 0.00	100.00 ± 0.00	100.00 ± 0.00	97.78 ± 2.22	10.00 ± 0.00	0.458	0.500	0.652
SGR (%/day)^c^	2.87 ± 0.07^a^	2.76 ± 0.08^a^ ^,^ ^b^	2.73 ± 0.03^a^ ^**,**^ ^b^	2.56 ± 0.05^a^ ^,^ ^b^	2.52 ± 0.13^b^	2.47 ± 0.06^b^	0.017	0.001	0.084
WG^c^ (%)	476.49 ± 22.89^a^	440.22 ± 24.77^a^ ^**,**^ ^b^	428.07 ± 9.24^a^ ^,^ ^b^	377.68 ± 15.15^a^ ^,^ ^b^	367.93 ± 35.31^b^	352.57 ± 17.86^b^	0.014	0.001	0.096
FI^c^ (%BW/day)	1.52 ± 0.04^a^	1.42 ± 0.03^a^ ^,^ ^b^	1.47 ± 0.02^a^ ^,^ ^b^	1.42 ± 0.05^a^ ^,^ ^b^	1.44 ± 0.03^a^ ^,^ ^b^	1.37 ± 0.03^b^	0.017	0.015	0.662
FER^c^	1.52 ± 0.04	1.59 ± 0.03	1.52 ± 0.03	1.51 ± 0.04	1.47 ± 0.04	1.53 ± 0.04	0.374	0.635	0.296
PER^c^	2.98 ± 0.08	3.09 ± 0.05	2.97 ± 0.05	2.93 ± 0.07	2.87 ± 0.08	2.94 ± 0.08	0.415	0.387	0.201
PR^c^	0.48 ± 0.01	0.48 ± 0.01	0.49 ± 0.01	0.49 ± 0.01	0.48 ± 0.01	0.49 ± 0.01	0.980	0.632	0.966
LR^c^	0.35 ± 0.01^**d**^	0.43 ± 0.01^c^	0.49 ± 0.01^c^	0.60 ± 0.02^b^	0.67 ± 0.01^a^	0.61 ± 0.01^b^	0.000	0.000	0.000
FY^c^(%)	69.16 ± 0.50^a^	67.28 ± 0.54^a^ ^,^ ^b^	66.49 ± 0.60^a^ ^,^ ^b^	66.36 ± 0.84^a^ ^,^ ^b^	66.80 ± 0.90^a^ ^,^ ^b^	65.61 ± 0.60^b^	0.009	0.000	0.960
Feed cost^c^ (USD/kg flesh)	1.28 ± 0.03^a^	1.21 ± 0.02^a^ ^,^ ^b^	1.24 ± 0.02^a^	1.19 ± 0.03^a^ ^**,**^ ^b^	1.17 ± 0.02^a^ ^**,**^ ^b^	1.10 ± 0.02^b^	0.005	0.001	0.027
CF^c^ (g/cm^3^)	3.42 ± 0.07	3.44 ± 0.05	3.34 ± 0.06	3.44 ± 0.05	3.44 ± 0.05	3.61 ± 0.11	0.722	0.955	0.625
HSI^c^ (%)	1.21 ± 0.05^a^ ^,^ ^b^	1.04 ± 0.04^b^	1.25 ± 0.06^a^ ^,^ ^b^	1.44 ± 0.11^a^	1.44 ± 0.08^a^	1.48 ± 0.11^a^	0.000	0.025	0.176
VSI^c^ (%)	5.38 ± 0.11	5.22 ± 0.09	5.39 ± 0.13	5.45 ± 0.10	5.55 ± 0.11	5.56 ± 0.18	0.316	0.488	0.052

a Data are means of triplicate. Means in the same row sharing the same superscript letter are not significantly different determined by Tukey’s test (*p* > 0.05).

b CAM0 was the control group, CAM15, CAM30, CAM45, CAM60 and CAM80 were replacement groups formulated by replacing 15%, 30%, 45%, 60% and 80% of fish meal in the control group.

c IBW, initial body weight; FBW, final body weight; SGR, specific growth rate; WGR, weight gain rate; FI, feed intake; FER, feed efficiency ratio; PER, protein efficiency ratio; PR, protein retention; LR, lipid retention; FY, flesh yield; CF, condition factor; HSI, hepatosomatic index; VSI, viscerosomatic index; Feed cost, converted from CHY to USD with the exchange rate of 1: 6.5.

### Body composition, profiles of amino acids and fatty acids in dorsal muscle

As compared with the control group, carcass moisture and protein were not significantly altered by dietary CAM (*p <* 0.05) (Table 7). With increasing dietary CAM, carcass lipid showed significant increase in linear pattern (*p <* 0.05). The essential amino acids content in muscle was not significantly affected by dietary CAM as compared with the control group (*p* > 0.05). As to fatty acids profile in muscle (Table 8), the levels of myristic acid (C14:0), total monounsaturated fatty acids and total n-6 polyunsaturated fatty acids showed significantly linear increase with increasing dietary CAM. While the content of n-3 polyunsaturated fatty acids, including EPA (C20:5 n-3), DHA (C22:6 n-3) and total n-3 PUFA, decreased with significantly linear and quadratic pattern with increasing dietary CAM (*p <* 0.05).

**TABLE 7:** Body composition and dorsal muscle amino acids profile (Means ± S.E.M)^a^.

	Diets^b^						Polynomial contrasts		
	CAM0	CAM15	CAM30	CAM45	CAM60	CAM80	*p*-value	Linear	Quadratic
Body composition									
Moisture (%)	76.66 ± 0.12	77.24 ± 0.54	76.69 ± 0.46	76.04 ± 0.02	75.71 ± 0.19	76.37 ± 0.10	0.070	0.134	0.086
Crude protein (% w.w^c^)	15.64 ± 0.07^a^ ^,^ ^b^	15.34 ± 0.06^b^	15.87 ± 0.09^a^	15.98 ± 0.10^a^	15.99 ± 0.07^a^	15.82 ± 0.07^a^	0.000	0.005	0.018
Crude lipid (% w.w^c^)	2.55 ± 0.11^d^	2.88 ± 0.02^d^	3.27 ± 0.04^c^	3.86 ± 0.07^a^ ^,^ ^b^	4.17 ± 0.15^a^	3.64 ± 0.06^b^ ^,^ ^c^	0.000	0.000	0.003
Ash (% w.w^c^)	3.39 ± 0.05^a^	3.34 ± 0.04^a^ ^,^ ^b^	3.26 ± 0.07^a^ ^,^ ^b^ ^,^ ^c^	3.06 ± 0.02^b^ ^,^ ^c^	3.10 ± 0.06^c^	3.15 ± 0.05^c^	0.001	0.000	0.192
Essential amino acids (%/dry matter)									
Threonine	4.15 ± 0.06^a^ ^,^ ^b^	4.19 ± 0.04^a^	4.19 ± 0.02^a^	4.03 ± 0.01^b^	4.08 ± 0.02^a^ ^,^ ^b^	4.20 ± 0.02^a^	0.010	0.497	0.804
Valine	4.38 ± 0.08^a,^ ^b^	4.43 ± 0.04^a^	4.48 ± 0.03^a^	4.30 ± 0.02^a^ ^,^ ^b^	4.23 ± 0.01^b^	4.46 ± 0.04^a^	0.005	0.650	0.365
Methionine	2.80 ± 0.05^a^ ^,^ ^b^	2.86 ± 0.02^a^	2.85 ± 0.02^a^	2.70 ± 0.02^b^	2.74 ± 0.01^a^ ^,^ ^b^	2.86 ± 0.02^a^	0.003	0.632	0.594
Isoleucine	4.27 ± 0.09^a^ ^,^ ^b^	4.33 ± 0.05^a^	4.37 ± 0.02^a^	4.18 ± 0.03^a^ ^,^ ^b^	4.07 ± 0.01^b^	4.31 ± 0.04^a^	0.009	0.259	0.171
Leucine	7.41 ± 0.13^a^ ^,^ ^b^ ^,^ ^c^	7.51 ± 0.07^a^ ^,^ ^b^	7.52 ± 0.05^a^	7.18 ± 0.03^c^	7.19 ± 0.02^b^ ^,^ ^c^	7.45 ± 0.05^a^ ^,^ ^b^ ^,^ ^c^	0.010	0.245	0.231
Phenylalanine	3.62 ± 0.05	3.62 ± 0.06	3.62 ± 0.01	3.54 ± 0.03	3.57 ± 0.03	3.66 ± 0.03	0.312	0.734	0.505
Lysine	7.99 ± 0.13^a^ ^,^ ^b^ ^,^ ^c^	8.13 ± 0.08^a^ ^,^ ^b^	8.14 ± 0.02^a^	7.79 ± 0.04^b^ ^,^ ^c^	7.76 ± 0.05^c^	8.05 ± 0.05^a^ ^,^ ^b^ ^,^ ^c^	0.008	0.307	0.142
Histidine	1.91 ± 0.02^a^ ^,^ ^b^ ^,^ ^c^	1.92 ± 0.02^a^ ^,^ ^b^	1.93 ± 0.02^a^	1.85 ± 0.01^b^ ^,^ ^c^	1.84 ± 0.01^c^	1.90 ± 0.01^a^ ^,^ ^b^ ^,^ ^c^	0.005	0.038	0.147
Arginine	5.46 ± 0.07^a^ ^,^ ^b^	5.52 ± 0.06^a^ ^,^ ^b^	5.52 ± 0.03^a^	5.31 ± 0.04^b^	5.34 ± 0.02^a^ ^,^ ^b^	5.50 ± 0.03^a^ ^,^ ^b^	0.015	0.376	0.406
Non-essential amino acids (%/dry matter)									
Aspartic acid	9.40 ± 0.14^a^ ^,^ ^b^	9.53 ± 0.06^a^	9.48 ± 0.06^a^ ^,^ ^b^	9.17 ± 0.05^b^	9.17 ± 0.02^b^	9.46 ± 0.06^a^ ^,^ ^b^	0.012	0.289	0.282
Serine	3.65 ± 0.04^a^ ^,^ ^b^	3.67 ± 0.03^a^ ^,^ ^b^	3.65 ± 0.02^a^ ^,^ ^b^	3.56 ± 0.02^b^	3.63 ± 0.02^a^ ^,^ ^b^	3.72 ± 0.03^a^	0.026	0.967	0.232
Glutamic acid	12.84 ± 0.25	13.06 ± 0.09	13.04 ± 0.05	12.51 ± 0.05	12.57 ± 0.04	12.97 ± 0.05	0.180	0.485	0.279
Glycine	4.24 ± 0.11^b^	4.27 ± 0.02^b^	4.26 ± 0.02^b^	4.28 ± 0.07^b^	4.42 ± 0.03^a^ ^,^ ^b^	4.57 ± 0.01^a^	0.007	0.005	0.004
Alanine	5.39 ± 0.07^b^	5.49 ± 0.03^a^ ^,^ ^b^	5.50 ± 0.04^a^ ^,^ ^b^	5.34 ± 0.04^b^	5.42 ± 0.03^a^ ^,^ ^b^	5.64 ± 0.09^a^	0.021	0.083	0.283
Cysteine	1.15 ± 0.14	1.29 ± 0.05	1.21 ± 0.07	1.15 ± 0.07	1.46 ± 0.08	1.35 ± 0.3	0.628	0.279	0.634
Tyrosine	3.24 ± 0.06	3.28 ± 0.05	3.28 ± 0.01	3.13 ± 0.01	3.15 ± 0.02	3.26 ± 0.03	0.034	0.295	0.522
Proline	2.47 ± 0.10	2.41 ± 0.06	2.42 ± 0.06	2.31 ± 0.04	2.52 ± 0.02	2.40 ± 0.04	0.281	0.478	0.490

a Data are means of triplicate. Means in the same row sharing the same superscript letter are not significantly different determined by Tukey’s test (*p* > 0.05).

b CAM0 was the control group, CAM15, CAM30, CAM45, CAM60 and CAM80 were replacement groups formulated by substituting 15%, 30%, 45%, 60% and 80% of fish meal in the control group.

c w.w, wet weight.

d This indicate that there is a significant difference in values between groups.

**TABLE 8:** Fatty acids profile in dorsal muscle of juvenile turbot (Means ± S.E.M)^a^.

Fatty acids profile/(% total fatty acids)	Diets^b^						Polynomial contrasts		
	CAM0	CAM15	CAM30	CAM45	CAM60	CAM80	*p*-value	Linear	Quadratic
C14:0	2.45 ± 0.11^b^	3.10 ± 0.29^a^ ^,^ ^b^	3.11 ± 0.40^a^ ^,^ ^b^	3.07 ± 0.15^a^ ^,^ ^b^	3.22 ± 0.16^a^ ^,^ ^b^	4.11 ± 0.04^a^	0.007	0.001	0.094
C16:0	23.46 ± 0.12^a^ ^,^ ^b^	22.51 ± 0.10^c^	22.58 ± 0.22^b^ ^,^ ^c^	22.42 ± 0.06^c^	23.72 ± 0.35^a^	22.65 ± 0.15^b^ ^,^ ^c^	0.001	0.049	0.008
C18:0	7.38 ± 0.01^a^	6.35 ± 0.26^a^	6.32 ± 0.45^a^	6.36 ± 0.18^a^	6.59 ± 0.07^a^	5.09 ± 0.02^b^	0.000	0.000	0.136
C24:0	2.08 ± 0.04^a^	2.22 ± 0.05^a^	2.15 ± 0.04^a^	1.98 ± 0.03^a^ ^,^ ^b^	1.76 ± 0.08^b^	1.98 ± 0.06^a^ ^,^ ^b^	0.001	0.013	0.001
∑ SFA^c^	35.37 ± 0.21^a^	34.18 ± 0.13^a^ ^,^ ^b^	34.16 ± 0.32^a^ ^,^ ^b^	33.83 ± 0.07^b^	35.28 ± 0.50^a^	33.82 ± 0.19^b^	0.004	0.007	0.135
C16:1	2.69 ± 0.12^b^	3.39 ± 0.30^a^ ^,^ ^b^	3.21 ± 0.41^a^ ^,^ ^b^	3.14 ± 0.12^a^ ^,^ ^b^	3.13 ± 0.11^a^ ^,^ ^b^	3.86 ± 0.05^a^	0.045	0.012	0.637
C17:1	0.43 ± 0.01^e^	0.66 ± 0.05^d^	0.79 ± 0.06^c^,^d^	0.90 ± 0.02^b^ ^,^ ^c^	1.09 ± 0.07^b^	1.46 ± 0.04^a^	0.000	0.000	0.000
C18:1,cis	13.01 ± 0.36^b^	13.64 ± 0.43^a^ ^,^ ^b^	13.79 ± 0.27^a^ ^,^ ^b^	14.29 ± 0.16^a^ ^,^ ^b^	14.74 ± 0.35^a^	15.17 ± 0.36^a^	0.007	0.000	0.040
C18:1,trans	3.13 ± 0.03	3.08 ± 0.08	3.04 ± 0.10	2.95 ± 0.01	2.99 ± 0.06	2.99 ± 0.04	0.412	0.064	0.792
C20:1	2.12 ± 0.07^c^	2.51 ± 0.16^b^ ^,^ ^c^	2.63 ± 0.27^b^ ^,^ ^c^	2.78 ± 0.07^b^ ^,^ ^c^	2.85 ± 0.10^b^	3.77 ± 0.11^a^	0.000	0.000	0.001
∑ MUFA^c^	21.39 ± 0.58^b^	23.28 ± 1.00^b^	23.46 ± 1.08^b^	24.07 ± 0.12^a^ ^,^ ^b^	24.81 ± 0.69^a^ ^,^ ^b^	27.25 ± 0.58^a^	0.003	0.000	0.029
C18:2 n-6	7.42 ± 0.04^c^	8.42 ± 0.26^b^ ^,^ ^c^	8.39 ± 0.44^b^ ^,^ ^c^	8.71 ± 0.09^b^	8.78 ± 0.15^b^	10.07 ± 0.04^a^	0.000	0.000	0.009
C20:2 n-6	0.51 ± 0.01^c^	0.58 ± 0.01^b^	0.60 ± 0.02^a^ ^,^ ^b^	0.57 ± 0.02^b^	0.57 ± 0.01^b^	0.65 ± 0.01^a^	0.000	0.000	0.460
C20:4 n-6	1.36 ± 0.04^a^	1.21 ± 0.05^a^ ^,^ ^b^	1.20 ± 0.10^a^ ^,^ ^b^	1.18 ± 0.04^a^ ^,^ ^b^	1.13 ± 0.04^a^ ^,^ ^b^	1.01 ± 0.02^b^	0.015	0.001	0.158
∑ n-6 PUFA^c^	9.29 ± 0.08^c^	10.22 ± 0.23^b^	10.19 ± 0.34^b^ ^,^ ^c^	10.46 ± 0.08^b^	10.48 ± 0.20^b^	11.72 ± 0.04^a^	0.000	0.000	0.007
C18:3 n-3	0.68 ± 0.01^b^	0.89 ± 0.07^b^	0.88 ± 0.10^b^	0.88 ± 0.03^b^	0.85 ± 0.01^b^	1.22 ± 0.02^a^	0.000	0.000	0.032
C20:5 n-3	7.11 ± 0.13^a^ ^,^ ^b^	7.55 ± 0.10^a^	7.01 ± 0.15^a^ ^,^ ^b^	6.77 ± 0.06^b^	6.03 ± 0.27^c^	6.42 ± 0.10^b^ ^,^ ^c^	0.000	0.001	0.012
C22:6 n-3	19.83 ± 0.59^a^	18.65 ± 0.54^a^ ^,^ ^b^	17.83 ± 0.83^a^ ^,^ ^b^ ^,c^	16.56 ± 0.21^b^ ^,^ ^c^ ^,^ ^d^	15.33 ± 0.81^c^ ^,^ ^d^	14.75 ± 0.52^d^	0.001	0.000	0.008
∑ n-3 PUFA^c^	27.62 ± 0.71^a^	27.09 ± 0.36^a^ ^,^ ^b^	25.72 ± 0.84^a^ ^,^ ^b^ ^,^ ^c^	24.22 ± 0.25^b^ ^,^ ^c^ ^,^ ^d^	22.21 ± 1.09^d^	22.39 ± 0.61^c,^ ^d^	0.000	0.000	0.003
∑ SFA/∑ PUFA	0.96 ± 0.03^a^ ^,^ ^b^	0.92 ± 0.01^b^	0.95 ± 0.01^b^	0.98 ± 0.01^a^ ^,^ ^b^	1.08 ± 0.06^a^	0.99 ± 0.02^a^ ^,^ ^b^	0.016	0.072	0.024
∑ n-3 PUFA/∑ n-6 PUFA	2.97 ± 0.05^a^	2.65 ± 0.10^a^ ^,^ ^b^	2.53 ± 0.16^b^	2.32 ± 0.03^b^ ^,^ ^c^	2.12 ± 0.06^c^ ^,^ ^d^	1.91 ± 0.05^d^	0.000	0.000	0.985

a Data are means of triplicate. Means in the same row sharing the same superscript letter are not significantly different determined by Tukey’s test (*p* > 0.05).

b CAM0 was the control group, CAM15, CAM30, CAM45, CAM60 and CAM80 were replacement groups formulated by replacing 15%, 30%, 45%, 60% and 80% fish meal in the control group.

c SFA, saturated fatty acids; MUFA, monounsaturated fatty acids; n-6 PUFA, n-6 polyunsaturated fatty acids; n-3 PUFA, n-3 polyunsaturated fatty acids. Some fatty acids with extremely low content are not listed.

d This indicate that there is a significant difference in values between groups.

### Apparent digestibility coefficient of feed

There were no significant differences in apparent digestibility coefficient (ADC) of dry matter and lipid between replacement groups and the control group (*p* > 0.05) (Table 9). While the ADC of protein showed a significantly linear increase pattern with increasing dietary CAM (*p <* 0.05). Also, the ADC of most essential amino acids, including threonine, valine, lysine, histidine and arginine, followed the same pattern as that of protein.

**TABLE 9:** Apparent digestibility coefficient of dry matter, protein, lipid and amino acids of the experimental diets (Means ± S.E.M)^a^.

	Diets^b^						Polynomial contrasts		
	CAM0	CAM15	CAM30	CAM45	CAM60	CAM80	*p*-value	Linear	Quadratic
Dry matter (%)	69.25 ± 0.37^a^ ^,^ ^b^	70.35 ± 0.29^a^	69.74 ± 0.27^a^ ^,^ ^b^	68.68 ± 0.16^b^	69.78 ± 0.33^a^ ^,^ ^b^	69.16 ± 0.43^a^ ^,^ ^b^	0.040	0.961	0.051
Protein (%)	91.03 ± 0.14^c^	92.02 ± 0.08^b^	92.73 ± 0.13^a^	91.71 ± 0.08^b^	92.89 ± 0.22^a^	92.93 ± 0.05^a^	0.000	0.000	0.267
Lipid (%)	76.08 ± 1.06	80.24 ± 0.61	78.91 ± 1.12	76.32 ± 0.70	78.29 ± 0.66	76.92 ± 1.66	0.352	0.190	0.783
Essential amino acids (%)									
Threonine	92.33 ± 0.29^b^	93.68 ± 0.14^a^	93.28 ± 0.07^a^	93.12 ± 0.13^a^ ^,^ ^b^	93.84 ± 0.20^a^	93.44 ± 0.21^a^	0.000	0.000	0.113
Valine	92.76 ± 0.28^c^	93.89 ± 0.05^a^	93.59 ± 0.09^a^ ^,^ ^b^	93.11 ± 0.16^b^ ^,^ ^c^	94.13 ± 0.11^a^	94.21 ± 0.11^a^	0.000	0.000	0.530
Methionine	94.39 ± 0.68	96.18 ± 0.51	95.65 ± 0.58	95.01 ± 0.62	94.67 ± 0.67	95.5 ± 0.60	0.358	0.343	0.162
Isoleucine	93.61 ± 0.21	94.67 ± 0.29	93.87 ± 0.23	93.31 ± 0.59	94.46 ± 0.25	94.78 ± 0.51	0.061	0.121	0.626
Leucine	94.93 ± 0.13	95.35 ± 0.08	95.11 ± 0.13	94.93 ± 0.19	95.23 ± 0.09	95.34 ± 0.08	0.082	0.079	0.922
Phenylalanine	93.60 ± 0.18	93.38 ± 0.35	93.24 ± 0.32	93.26 ± 0.22	93.81 ± 0.20	94.28 ± 0.3	0.095	0.337	0.011
Lysine	96.72 ± 0.15^b^	97.21 ± 0.06^a^ ^,^ ^b^	97.02 ± 0.12^a^ ^,^ ^b^	96.82 ± 0.14^a^ ^,^ ^b^	97.19 ± 0.11^a^ ^,^ ^b^	97.33 ± 0.13^a^	0.014	0.006	0.741
Histidine	94.68 ± 0.10^c^	95.25 ± 0.04^a^ ^,^ ^b^ ^,^ ^c^	95.12 ± 0.02^b^ ^,^ ^c^	95.10 ± 0.05^b^ ^,^ ^c^	95.66 ± 0.17^a^ ^,^ ^b^	95.95 ± 0.35^a^	0.001	0.000	0.033
Arginine	94.26 ± 0.24^b^	95.34 ± 0.03^a^	95.17 ± 0.06^a^	95.14 ± 0.04^a^	95.52 ± 0.11^a^	95.72 ± 0.16^a^	0.000	0.000	0.982
Non-essential amino acids (%)									
Aspartic acid	92.57 ± 0.37^c^	93.90 ± 0.24^a^ ^,^ ^b^	93.66 ± 0.23^b^	93.75 ± 0.16^a^ ^,^ ^b^	94.75 ± 0.17^a^	94.76 ± 0.2^a^	0.000	0.000	0.147
Serine	92.70 ± 0.34^b^	93.53 ± 0.13^a^	93.13 ± 0.13^a^ ^b^	93.01 ± 0.11^a^ ^,^ ^b^	93.34 ± 0.09^a^ ^,^ ^b^	93.31 ± 0.13^a^ ^,^ ^b^	0.051	0.020	0.260
Glutamic acid	94.55 ± 0.16^b^ ^,^ ^c^	95.20 ± 0.08^a^	95.00 ± 0.03^a^	94.99 ± 0.05^a^ ^,^ ^b^	95.06 ± 0.10^a^	94.46 ± 0.11^c^	0.000	0.107	0.000
Glycine	93.74 ± 0.24	94.38 ± 0.07	94.08 ± 0.10	93.82 ± 0.11	93.78 ± 0.46	94.18 ± 0.21	0.365	0.446	0.324
Alanine	94.26 ± 0.16^c^	94.85 ± 0.09^a^	94.74 ± 0.05^a^ ^,^ ^b^	94.37 ± 0.08^b^ ^,^ ^c^	94.50 ± 0.09^a^ ^,^ ^b^ ^,^ ^c^	94.39 ± 0.06^b^ ^,^ ^c^	0.002	0.183	0.000
Cysteine	83.98 ± 1.51	84.56 ± 0.57	84.12 ± 0.98	83.97 ± 0.94	84.72 ± 1.24	86.75 ± 0.54	0.413	0.227	0.187
Tyrosine	95.54 ± 0.22^b^	95.41 ± 0.33^b^	95.48 ± 0.28^b^	95.62 ± 0.23^b^	96.23 ± 0.11^a^ ^,^ ^b^	96.70 ± 0.15^a^	0.005	0.009	0.001
Proline	88.18 ± 0.12^a^ ^,^ ^b^	89.09 ± 0.21^a^	88.93 ± 0.26^a^	88.38 ± 0.41^a^ ^,^ ^b^	88.43 ± 0.29^a^ ^,^ ^b^	87.31 ± 0.32^b^	0.005	0.394	0.000

a Data are means of triplicate. Means in the same row sharing the same superscript letter are not significantly different determined by Tukey’s test (*p* > 0.05).

b CAM0 was the control group, CAM15, CAM30, CAM45, CAM60 and CAM80 were replacement groups formulated by replacing 15%, 30%, 45%, 60% and 80% of fish meal in the control group.

### Serum and hepatic biochemical index

The serum GPT activity was significantly linear decreased with increasing dietary CAM (*p* < 0.05) (Table 10). Also, the GOT activity in serum was significantly reduced in replacement groups (except group CAM60) as compared with the control group (*p* < 0.05). Compared with the control group, the serum HDL-C content tended to be increased in replacement groups, and significant differences were observed when the levels of CAM protein replacing fish meal protein were no more than 30% (*p* < 0.05). The serum TG content was significantly reduced in fish fed diet with CAM (*p* < 0.05). While the hepatic TG content was significantly increased in fish fed with diets CAM60 and CAM80 as compared with the control group (*p* < 0.05).

**TABLE 10:** Serum and hepatic biochemical index (Means ± S.E.M)^a^.

	Diets^b^						Polynomial contrasts		
	CAM0	CAM15	CAM30	CAM45	CAM60	CAM80	*p*-value	Linear	Quadratic
Serum									
GPT^c^(IU/L)	10.89 ± 0.10^a^	10.93 ± 0.03^a^	10.42 ± 0.17^a^	10.07 ± 0.27^a^ ^,^ ^b^	9.42 ± 0.27^b^ ^,^ ^c^	8.97 ± 0.23^c^	0.000	0.000	0.880
GOT^c^(IU/L)	3.75 ± 0.13^a^	2.34 ± 0.31^b^ ^,^ ^c^	2.28 ± 0.11^b^ ^,^ ^c^	2.07 ± 0.05^c^	3.13 ± 0.19^a^ ^,^ ^b^	2.61 ± 0.26^b^ ^,^ ^c^	0.000	0.101	0.001
HDL-C^c^ (mmol/L)	0.87 ± 0.03^b^	1.12 ± 0.02^a^	1.08 ± 0.02^a^	0.99 ± 0.02^a^ ^,^ ^b^	1.06 ± 0.08^a^ ^,^ ^b^	1.00 ± 0.07^a^ ^,^ ^b^	0.016	0.055	0.009
LDL-C^c^ (mmol/L)	0.72 ± 0.06^a^ ^,^ ^b^	0.62 ± 0.02^a^ ^,^ ^b^ ^,^ ^c^	0.56 ± 0.02^b^ ^,^ ^c^	0.77 ± 0.05^a^	0.50 ± 0.02^c^	0.57 ± 0.06^b^ ^,^ ^c^	0.003	0.017	0.725
T-CHO^c^ (mmol/L)	2.59 ± 0.18^a^	1.72 ± 0.09^b^	1.71 ± 0.03^b^	2.09 ± 0.12^b^	1.89 ± 0.04^b^	1.77 ± 0.08^b^	0.000	0.108	0.330
TG^c^ (umol/L)	1.49 ± 0.04^a^	0.55 ± 0.02^c^	0.71 ± 0.03^c^	1.14 ± 0.08^b^	1.05 ± 0.08^b^	0.72 ± 0.03^c^	0.000	0.082	0.043
Glucose (mmol/L)	1.67 ± 0.15^a^	0.20 ± 0.01^b^	0.23 ± 0.04^b^	0.26 ± 0.21^b^	0.39 ± 0.01^b^	0.45 ± 0.07^b^	0.000	0.034	0.000
Liver									
TG (umol/L)	13.62 ± 0.16^c^	10.67 ± 0.18^d^	12.99 ± 0.37^c^	10.94 ± 0.28^d^	14.95 ± 0.22^b^	16.57 ± 0.45^a^	0.001	0.005	0.001
T-CHO (umol/g liver)	5.62 ± 0.20^a^	5.05 ± 0.18^a^ ^,^ ^b^	4.54 ± 0.11^b^	4.68 ± 0.25^b^	4.42 ± 0.06^b^	5.16 ± 0.18^a^ ^,^ ^b^	0.001	0.015	0.039
Glucose (umol/g liver)	2.50 ± 0.06^b^	2.95 ± 0.25^a^ ^,^ ^b^	3.18 ± 0.10^a^	3.16 ± 0.14^a^	3.18 ± 0.16^a^	3.19 ± 0.10^a^	0.022	0.001	0.546

a Data are means of triplicate. Means in the same row sharing the same superscript letter are not significantly different determined by Tukey’s test (*p* > 0.05).

b CAM0 was the control group, CAM15, CAM30, CAM45, CAM60 and CAM80 were replacement groups formulated by replacing 15%, 30%, 45%, 60% and 80% of fish meal in the control group.

c GOT, glutamic oxalacetic transaminase; GPT, glutamic-pyruvic transaminase; HDL-C, high-density lipoprotein cholesterol; LDL-C, high-density lipoprotein cholesterol; T-CHO, total cholesterol; TG, triglycerides.

^d^ This indicate that there is a significant difference in values between groups.

### Hepatic morphology

**FIGURE 1:**
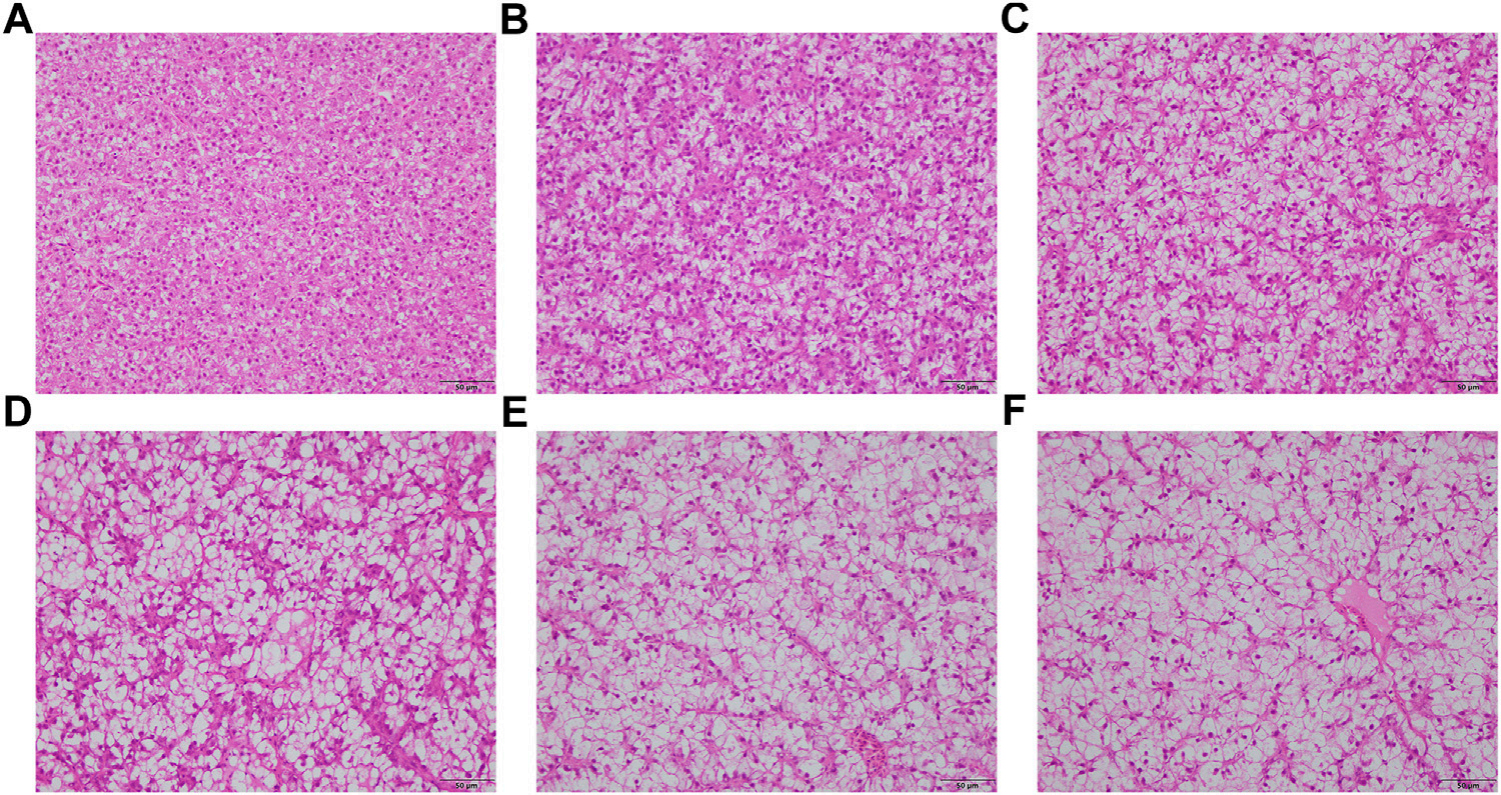
Representative liver sections (stained by haematoxylin and eosin) of juvenile turbot fed with different diets (Scale bars, 50 μm). CAM0 **(A)** was the control group, CAM15 **(B)**, CAM30 **(C)**, CAM45 **(D)**, CAM60 **(E)** and CAM80 **(F)** were replacement groups formulated by replacing 15%, 30%, 45%, 60% and 80% fish meal in the control group.

As the levels of CAM substituted for fish meal increased, hepatocyte vacuolization became more and more severe, accompanied by an increase in cellular size and nuclear deviation (Figure 1). Meanwhile, the nuclear atrophy and disappearance were most serious in fish fed with diets CAM60 and CAM80.

### Relative expression of genes related to peptide and amino acids transporters in intestine

**FIGURE 2:**
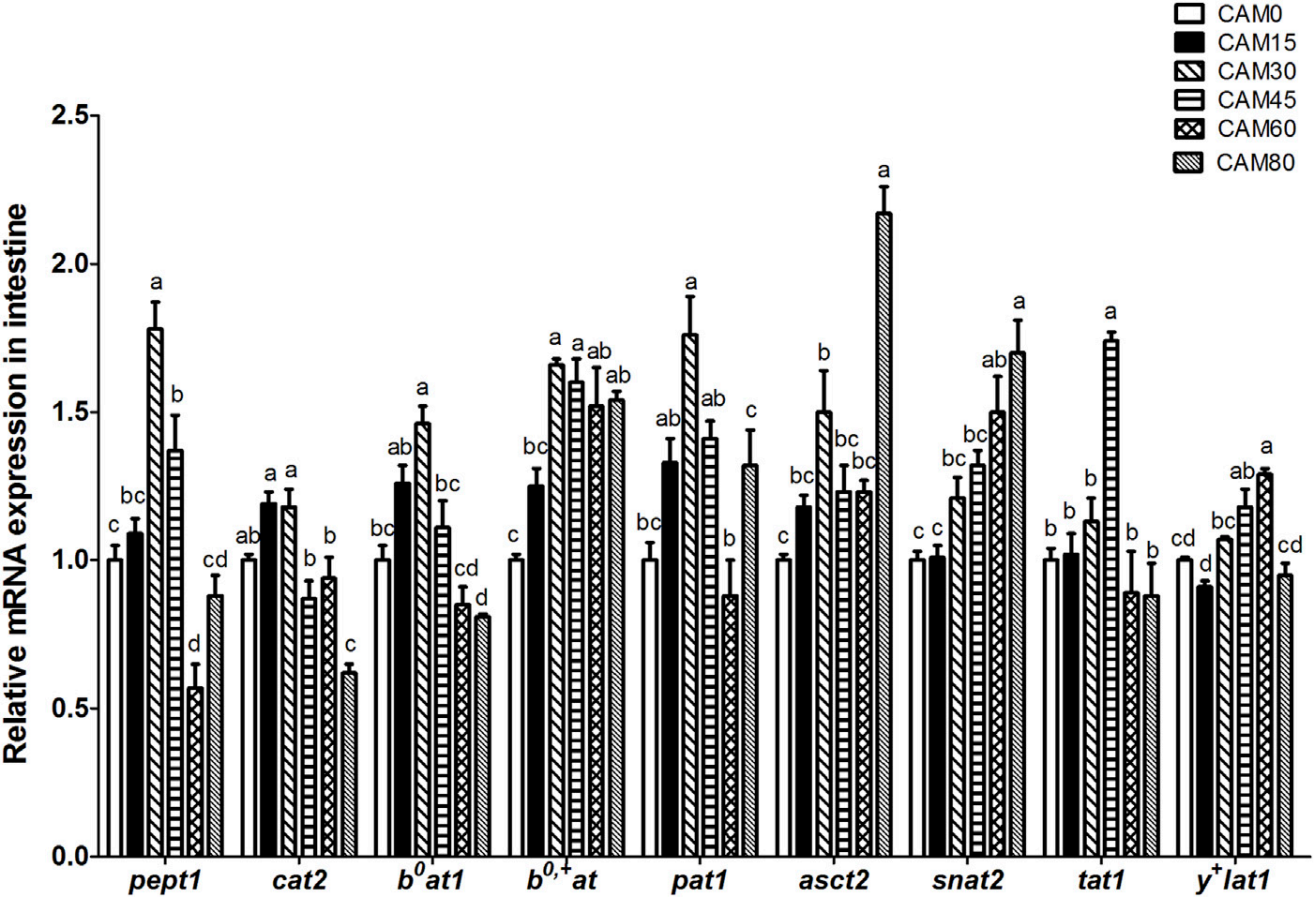
Relative mRNA expression of peptide and amino acids transporters in intestine of turbot fed different diets. Data are presented as means ± S.E.M (*n* = 3). Columns sharing the same superscript letter or absence of superscripts are not significantly different determined by Tukey’s test (*p* > 0.05). Transcriptional levels were normalized by the reference gene of RNA polymerase II subunit D (*rpsd*). *pept1*, peptide transporter 1; *cat2*, cationic amino acid transporter 2; *b* ^*0*^ *at1*, B^0^-type amino acid transporter 1; *b* ^*0,+*^ *at*, b^0,+^-type amino acid transporter; *pat1*, proton-coupled amino acid transporter 1; *asct2*, system ASC amino acid transporter-2; *snat2*, sodium-coupled neutral amino acid transporter 2; *tat1*, T-type amino acid transporter 1; *y* ^*+*^ *lat1*, system y^+^L amino acid transporter 1. Polynomial contrasts analysis: *pept1*: *P*_value_ = 0.000, *P*_Linear_ = 0.354, *P*_Quadratic_ = 0.000; *cat2*: *P*_value_ = 0.000, *P*_Linear_ = 0.001, *P*_Quadratic_ = 0.023; *b* ^*0*^ *at1*: *P*_value_ = 0.000, *P*_Linear_ = 0.166, *P*_Quadratic_ = 0.000; *b* ^*0,+*^ *at*: *P*_value_ = 0.000, *P*_Linear_ = 0.000, *P*_Quadratic_ = 0.337; *pat1*: *P*_value_ = 0.000; *P*_Linear_ = 0.073, *P*_Quadratic_ = 0.001; *asct2*: *P*_value_ = 0.000, *P*_Linear_ = 0.000, *P*_Quadratic_ = 0.000; *snat2*: *P*_value_ = 0.001, *P*_Linear_ = 0.004, *P*_Quadratic_ = 0.927; *tat1*: *P*_value_ = 0.000, *P*_Linear_ = 0.381, *P*_Quadratic_ = 0.007; *y* ^*+*^ *lat1*: *P*_value_ = 0.000, *P*_Linear_ = 0.008, *P*_Quadratic_ = 0.445.

With increasing dietary CAM, the gene expression of peptide and amino acids transporters in intestine, such as peptide transporter 1 (*pept1*), cationic amino acid transporter (*cat2*), B^0^-type amino acid transporter 1 (*b* ^*0*^ *at1*), proton-coupled amino acid transporter 1 (*pat1*), system ASC amino acid transporter-2 (*asct2*) and T-type amino acid transporter 1 (*tat1*), increased first and then decreased with significantly quadratic pattern (*p <* 0.05), and peak value was observed in fish fed with diet CAM30 or CAM45 (Figure 2).

### Relative expression of genes related to protein metabolism in liver and muscle

**FIGURE 3:**
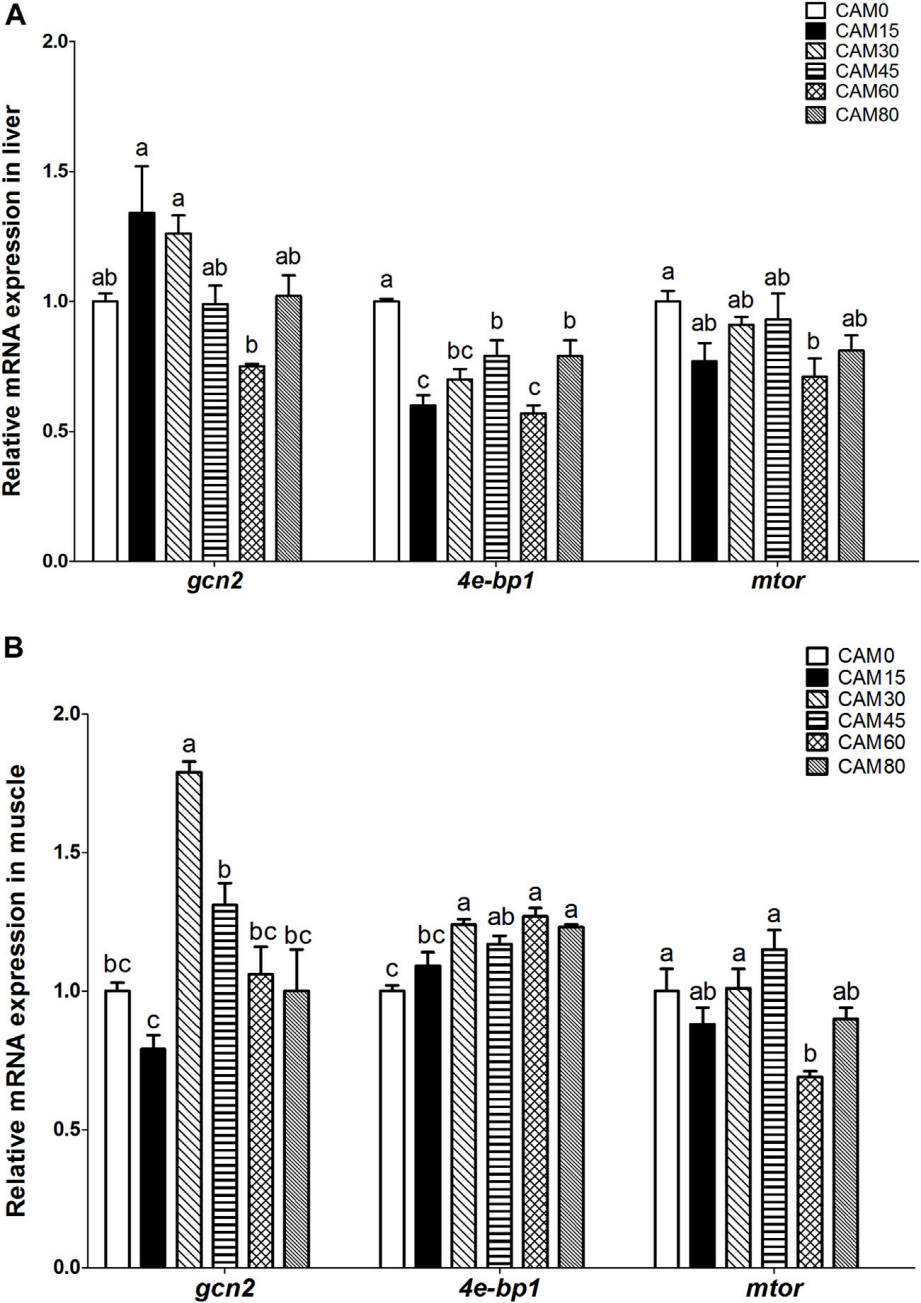
Relative mRNA expression related to protein metabolism in liver **(A)** and muscle **(B)** of juvenile turbot fed different diets. Data are presented as means ± S.E.M (*n* = 3). Columns sharing the same superscript letter or absence of superscripts are not significantly different determined by Tukey’s test (*p* > 0.05). Transcriptional levels were normalized by the reference gene of RNA polymerase II subunit D (*rpsd*). *gcn2*, general control nonderepressible 2; *mtor*, mammalian target of rapamycin; *4e-bp1*, eukaryotic initiation factor 4E-binding protein 1. Polynomial analysis in **(A)**: *gcn2*: *P*_value_ = 0.003, *P*_Linear_ = 0.454, *P*_Quadratic_ = 0.002; *4e-bp1*: *P*_value_ = 0.000, *P*_Linear_ = 0.000, *P*_Quadratic_ = 0.001; *mtor*: *P*_value_ = 0.038, *P*_Linear_ = 0.123, *P*_Quadratic_ = 0.898. Polynomial analysis in **(B)**: *gcn2*: *P*_value_ = 0.000, *P*_Linear_ = 0.154, *P*_Quadratic_ = 0.016; *4e-bp1*: *P*_value_ = 0.000, *P*_Linear_ = 0.054, *P*_Quadratic_ = 0.599; *mtor*: *P*_value_ = 0.001, *P*_Linear_ = 0.114, *P*_Quadratic_ = 0.254.

**FIGURE 4:**
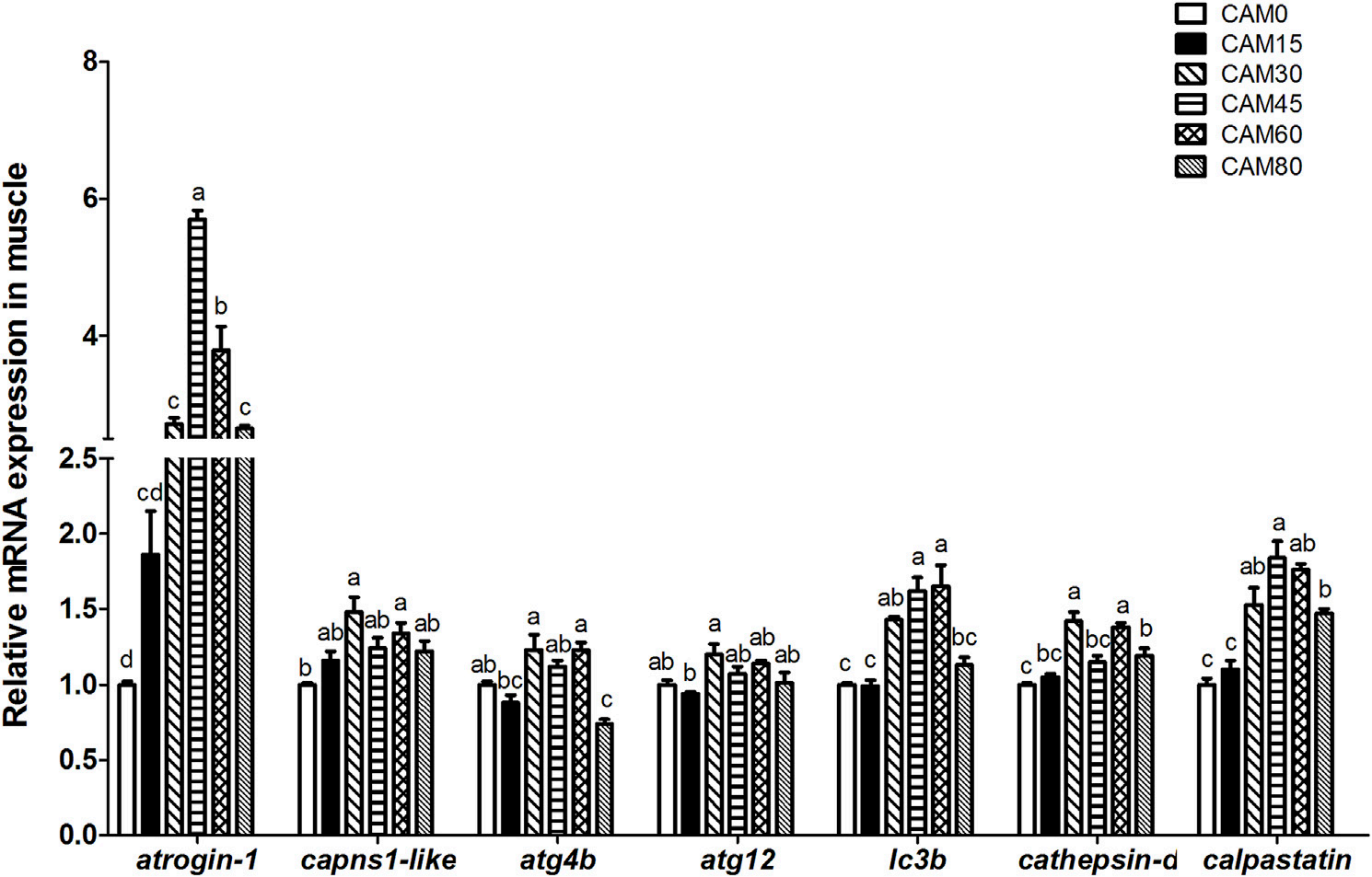
Relative mRNA expression related to protein degradation in muscle of juvenile turbot fed different diets. Data are presented as means ± S.E.M (*n* = 3). Columns sharing the same superscript letter or absence of superscripts are not significantly different determined by Tukey’s test (*p* > 0.05). Transcriptional levels were normalized by the reference gene of RNA polymerase II subunit D (*rpsd*). *capns1-like,* calpain small subunit 1-like; *atg4b*, autophagy related 4B cysteine peptidase. *atg12*, autophagy related 12 homolog. Polynomial analysis: *atrogin-1*: *P*_value_ = 0.000, *P*_Linear_ = 0.127, *P*_Quadratic_ = 0.000; *capns1-like*: *P*_value_ = 0.004, *P*_Linear_ = 0.003, *P*_Quadratic_ = 0.029; *atg4b*: *P*_value_ = 0.000, *P*_Linear_ = 0.936, *P*_Quadratic_ = 0.013; *atg12*: *P*_value_ = 0.008, *P*_Linear_ = 0.140, *P*_Quadratic_ = 0.749; *lc3b*: *P*_value_ = 0.000, *P*_Linear_ = 0.004, *P*_Quadratic_ = 0.000; *cathepsin-d*: *P*_value_ = 0.001, *P*_Linear_ = 0.000, *P*_Quadratic_ = 0.623; *calpastatin*: *P*_value_ = 0.001, *P*_Linear_ = 0.000, *P*_Quadratic_ = 0.004.

With increasing dietary CAM, the expression of general control nonderepressible 2 (*gcn2*) in liver and muscle tended to be first up-regulated and then down-regulated with significantly quadratic pattern (*p <* 0.05) (Figure 3A). In liver and muscle, the mammalian target of rapamycin (*mtor*) gene expression in replacement groups was not significantly different from that in the control group (*p* > 0.05), except for group CAM60 where the *mtor* gene expression was significantly lower than the control group (*p <* 0.05) (Figure 3B). The gene expression of eukaryotic initiation factor 4E-binding protein 1 (*4e-bp1*) in liver was significantly reduced in replacement groups as compared with the control group (*p <* 0.05). In muscle, *4e-bp1* expression in replacement groups was increased as compared with the control group, and significant differences were observed when the replacement levels reached 30% (*p <* 0.05). With the increase of dietary CAM, genes related protein degradation in muscle, such as *atrogin-1*, calpain small subunit 1-like (*capns1-like*), autophagy related 4B cysteine peptidase (*atg4b*), microtubule associated protein 1 light chain 3 beta (*lc3b*) and *calpastatin*, increased first and then decreased with significantly quadratic pattern (*p* < 0.05), and the peak value appeared in group CAM30 or CAM45 (Figure 4).

### Relative expression of genes related to lipid metabolism in liver

**FIGURE 5:**
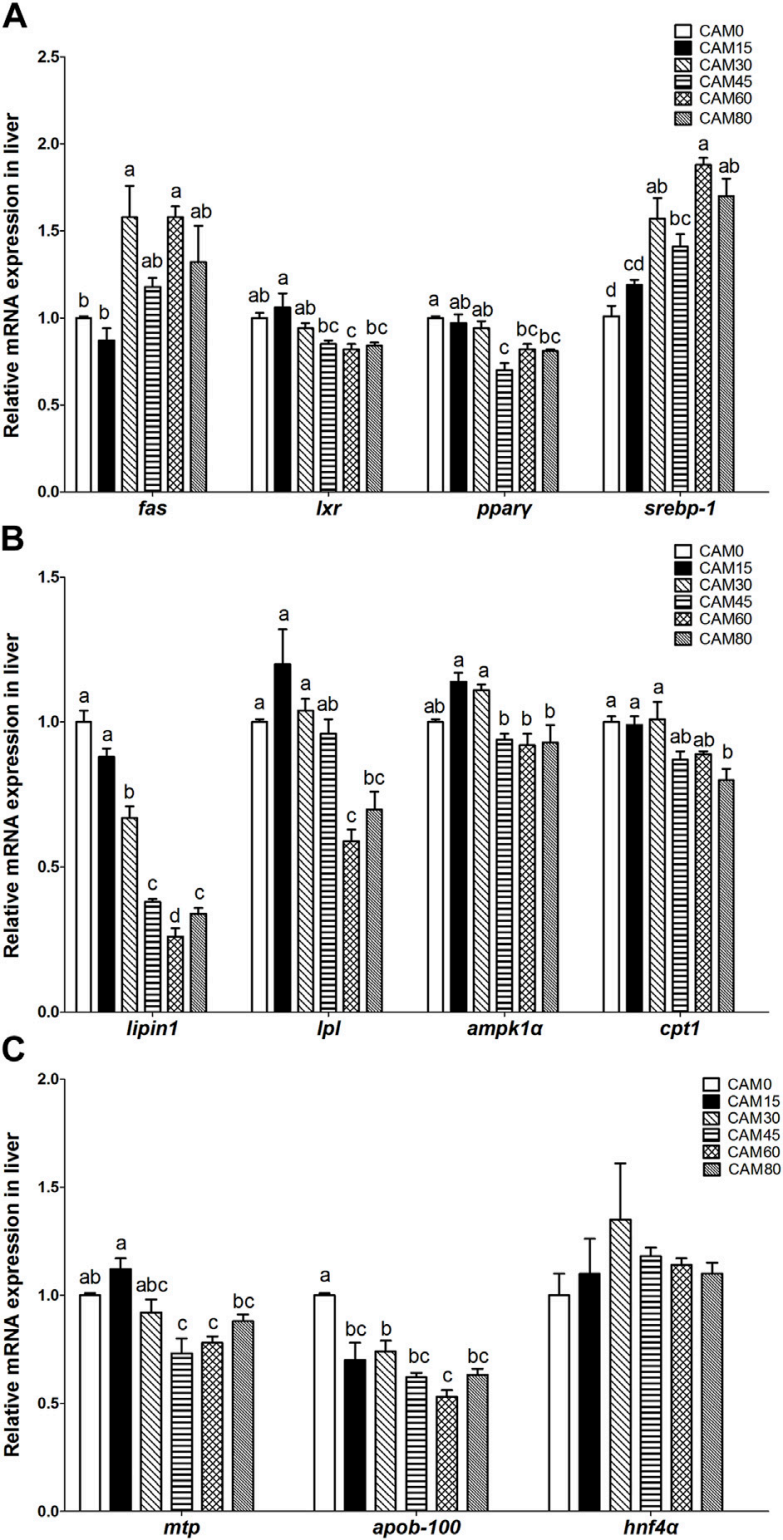
Relative mRNA expression related to lipid metabolism in liver of juvenile turbot fed different diets. lipogenesis related genes **(A)**; lipid oxidation related genes **(B)**; lipid transport related genes **(C)**. Data are presented as means ± S.E.M (*n* = 3). Columns sharing the same superscript letter or absence of superscripts are not significantly different determined by Tukey’s test (*p* > 0.05). Transcriptional levels were normalized by the reference gene of RNA polymerase II subunit D (*rpsd*). *as*, fatty acid synthase; *lxr*, liver X receptor; *pparγ*, peroxisome proliferator-activated receptor γ; *srebp-1*, sterol-regulatory element binding protein-1; *lpl*, lipoprotein lipase; *ampk1α*, adenosine monophosphate activated protein kinase1α;*cpt1*, carnitine palmitoyl transferase 1; *mtp*, mitochondrial trifunctional protein; *apob-100*, apolipoprotein B-100; *hnf4α*, hepatocyte nuclear factor 4α. Polynomial analysis: *fas*: *P*_value_ = 0.003, *P*_Linear_ = 0.009, *P*_Quadratic_ = 0.338; *lxr*: *P*_value_ = 0.003, *P*_Linear_ = 0.002, *P*_Quadratic_ = 0.012; *pparγ*: *P*_value_ = 0.000, *P*_Linear_ = 0.000, *P*_Quadratic_ = 0.085; *srebp-1*: *P*_value_ = 0.000, *P*_Linear_ = 0.000, *P*_Quadratic_ = 0.026; *lipin1*: *P*_value_ = 0.000, *P*_Linear_ = 0.004, *P*_Quadratic_ = 0.100; *lpl*: *P*_value_ = 0.000, *P*_Linear_ = 0.001, *P*_Quadratic_ = 0.018; *ampk1α*: *P*_value_ = 0.000, *P*_Linear_ = 0.060, *P*_Quadratic_ = 0.021; *cpt1*: *P*_value_ = 0.001, *P*_Linear_ = 0.001, *P*_Quadratic_ = 0.003; *mtp*: *P*_value_ = 0.000, *P*_Linear_ = 0.002, *P*_Quadratic_ = 0.022; *apob-100*: *P*_value_ = 0.000, *P*_Linear_ = 0.000, *P*_Quadratic_ = 0.602; *hnf4α*: *P*_value_ = 0.628, *P*_Linear_ = 0.413, *P*_Quadratic_ = 0.352.

The expression of genes related to lipogenesis (Figure 5A), such as fatty acid synthase (*fas*) and sterol-regulatory element binding protein-1 (*srebp-1*), tended to be increased in replacement groups as compared with the control group, and significant differences occurred when the substitution levels reached 30% (*p <* 0.05). In terms of genes related to lipid oxidation (Figure 5B), *lipin1* was significantly linear reduced with increasing dietary CAM (*p* < 0.05). Also, the expression of lipoprotein lipase (*lpl*) and carnitine palmitoyl transferase 1 (*cpt1*) was significantly reduced as compared with the control group when the replacement levels reached 60% and 80%, respectively (*p* < 0.05). For genes related to lipid transport (Figure 5C), apolipoprotein B-100 (*apob-100*) was significantly linear reduced with increasing dietary CAM (*p* < 0.05), and the expression of apolipoprotein B-100 (*mtp*) was significantly reduced as compared with the control group when the replacement levels reached 45% (*p* < 0.05).

## Discussion

In the present study, the survival rate (from 97.78 to 100%) was not significantly affected by dietary CAM. Coupled with the absence of disease in growth trial, which initially indicated the safety of CAM applied in aquafeeds. Besides, no significant differences were observed in growth parameters (FBW, SGR and WGR) among fish fed with diets CAM0, CAM15, CAM30 and CAM45, which suggested that dietary fish meal protein could be well replaced with CAM protein up to 45% without negative effects on growth performance of juvenile turbot. This resembled previous studies on black sea bream and largemouth bass, where CAM protein could replace 58.2% and 42.8% of dietary fish meal protein, respectively, without compromising growth performance (Chen et al., 2019; Yang P. et al., 2021). In terms of economic benefits, the flesh yield (FY) was not significantly reduced until the level of CAM protein substitution for fish meal protein reached 80%. Besides, feed cost showed significantly linear decrease with increasing dietary CAM, suggesting high commercial potential of CAM applied in aquafeeds production. On the whole, CAM is a promising protein in aquafeeds in terms of impacts on growth performance and feed cost.

Nevertheless, significantly reduced growth performance in fish fed diets with excessive levels of CAM was also observed in this study, which is worthy of further analysis in order to provide guidance for improving the CAM quality in the future. According to previous studies, the limitations of high levels of alternative protein sources applied in aquafeeds can be ascribed to poor palatability (Hauptman et al., 2014), low digestibility (Mundheim et al., 2004), imbalanced amino acids profile (Berge et al., 2005) as well as negative effects on amino acids transport and protein metabolism (Xu et al., 2017; Xie et al., 2019). Evidences in literature have reported that high levels of nucleic acids and minerals, especially free purine, iron and copper, in single-cell protein would reduce appetite (Rumsey et al., 1991; Aas et al., 2006). In view of the compromised appetite, the feed intake would inevitably be reduced. In this study, the feed intake (FI) was significantly linear decreased with increasing dietary CAM, which was consistent with early studies on black sea bream (Chen et al., 2019) and pacific white shrimp (Jiang et al., 2021). This indicated that CAM may have negative effect on feed palatability. As to the feed digestibility, the apparent digestibility coefficient (ADC) of dry matter and lipid was not significantly affected by dietary CAM, which was in line with study on largemouth bass (Yang P. et al., 2021). Besides, the ADC of protein and most essential amino acids tended to be increased by dietary CAM. These results demonstrated that dietary CAM was unlikely to compromise feed digestibility. Apart from that, the compromised growth performance is always connected with the imbalance of feed amino acids profile when fish fed diets with single-cell protein (Davies and Wareham, 1988; Berge et al., 2005). Given the insufficient arginine and histidine content in CAM, diets containing CAM were supplemented with L-arginine and L-histidine to meet the requirements reported for turbot (Kaushik, 1998), indicating dietary amino acids profile might not limit the growth performance. But considering the reduced feed intake and the leakage of provided amino acids, it cannot be directly concluded that dietary amino acids actually utilized can meet the requirement of juvenile turbot, further analysis needs to be performed next.

Amino acids are absorbed mainly in the form of peptide-bound and free amino acids in intestine (Krehbiel and Matthews, 2003; Stelzl et al., 2016), which means that intestinal peptide and amino acids transporters play vital roles in the dietary nutrients absorption. Previous studies have reported that gene expression of peptide and amino acids transporters in intestine was up-regulated when dietary fish meal was replaced with animal and vegetable protein sources (Xu et al., 2017; Yang X. et al., 2021). And the expression of peptide transporter could be a negative feedback regulation, in which the intestine would increase the absorption of peptide by increasing the expression of peptide transporter in a condition of undernutrition (Verri et al., 2011). In the present study, the trichloroacetic acid (TCA)-soluble protein content (reflecting the content of small peptide and free amino acids) of CAM (5.43%) was lower than that of fish meal (15.84%). And the gene expression of *pept1* in intestine was up-regulated when the replacement levels of dietary fish meal with CAM were no more than 45%. Also, the detected gene expression of amino acids transporters, including *cat2*, *b* ^*0*^ *at1*, *b* ^*0,+*^ *at*, *pat1*, *asct2, snat2* and *tat1*, exhibited similar pattern to that of *pept1*. These results initially implied that diet containing CAM may provide insufficient small peptide and free amino acids for juvenile turbot. As to the reduced gene expression of peptide and amino acids transporters in fish fed diets with high levels of CAM, which may be a adaptive response to a chronic status of amino acids deprivation as described by Orozco et al. (2017).

Amino acids balance and protein metabolism in organism are regulated by a combination of the mammalian target of rapamycin (mTOR) and the general control nonderepressible 2 (GCN2) signaling pathways, which would ultimately affect growth performance (Wang and Proud, 2006; Liu et al., 2019). In a condition of sufficient amino acids, the mTOR signaling pathway would be in a dominant role. While amino acids deficiency would activate GCN2 signaling pathway, which inhibits protein synthesis and facilitates the process of amino acids transport and synthesis (Liu et al., 2019). In the present study, the gene expression of *mtor* in liver and muscle was not significantly affected by dietary CAM in general. But the *gcn2* expression in liver and muscle tended to be first up-regulated and then down-regulated with significantly quadratic pattern, which was similar to the trend as peptide and amino acids transporters in intestine. Likewise, the expression of genes related to protein degradation in muscle also followed the pattern of increasing first and then decreasing. Therefore, we speculated that CAM-containing diets may provide insufficient amino acids for juvenile turbot, so that the organisms improved amino acids transport and protein degradation for the balance of amino acids. As for the decreasing trend of *gcn2* and gene related to protein degradation, it may also indicate adaptation of turbot to a chronic amino acids deficient status as discussed above. Therefore, the insufficient supply of small peptide and free amino acids resulted from dietary CAM could compromise the protein metabolism, and then lead to growth reduction of turbot.

Apart from growth performance, the increased lipid retention (LR), carcass lipid as well as altered muscle fatty acids profile in the present study suggested that lipid metabolism of turbot was also affected by dietary CAM. Stubhaug et al. (2007) argued that an imbalanced dietary fatty acids profile, especially a reduction in long-chain polyunsaturated fatty acids, would reduce β-oxidation and increase lipid deposition. The fatty acids composition of CAM was dominated by C14:0 and C16:0, and is extremely deficient in polyunsaturated fatty acids, which may cause lipid deposition in body. Liver is the center of lipid metabolism (Rui, 2011). A series of studies have shown that replacing fish oil with vegetable oil (deficient in long-chain polyunsaturated fatty acids) would lead to excessive lipid deposition in fish liver (Caballero et al., 2004; Castro et al., 2016; Torrecillas et al., 2017). Also, high dietary CAM resulted in excessive lipid deposition in liver, evidenced by increased TG content and hepatosomatic index. Besides, the hepatocyte size and vacuolization were also increased in fish fed diets with high levels of CAM, which resembled the study performed by Castro et al. (2016) and Torrecillas et al. (2017). It is worth noting that the activity of GOT and GPT in serum was not increased in this study, implying that hepatic lipid deposition caused by CAM may not reached the extent of liver damage. In this study, hepatic genes related to lipogenesis, such as *fas* and *srebp-1*, tended to be significantly up-regulated as the levels of CAM substitution for fish meal reached 30%, while genes related to lipid oxidation and lipid transport in liver showed the opposite trend. This was similar to previous studies on replacing dietary fish oil with vegetable oil, such as soybean oil and linseed oil (Peng et al., 2014; Wang et al., 2016; Yu et al., 2019). These results demonstrated the imbalanced fatty acids of CAM would result in excessive lipid deposition by increasing lipid synthesis and reducing lipid oxidation.

In conclusion, CAM is a promising alternative protein, which can replace 45% fish meal protein in diet for juvenile turbot without significantly adverse effects on growth performance. But excessive dietary CAM would result in significant growth reduction, and excessive lipid deposition may also occur in fish fed diets with high levels of CAM.

## Data Availability

The original contributions presented in the study are included in the article/Supplementary Materials, further inquiries can be directed to the corresponding author.
